# Drug Delivery Strategies and Biomedical Significance of Hydrogels: Translational Considerations

**DOI:** 10.3390/pharmaceutics14030574

**Published:** 2022-03-05

**Authors:** Neha Raina, Rakesh Pahwa, Jaydeep Bhattacharya, Alok K. Paul, Veeranoot Nissapatorn, Maria de Lourdes Pereira, Sonia M. R. Oliveira, Karma G. Dolma, Mohammed Rahmatullah, Polrat Wilairatana, Madhu Gupta

**Affiliations:** 1 Department of Pharmaceutics, Delhi Pharmaceutical Sciences and Research University, New Delhi 110017, India; rainanehaceutics@gmail.com; 2 Institute of Pharmaceutical Sciences, Kurukshetra University, Kurukshetra 136119, India; rakesh_pahwa2407@yahoo.co.in; 3 School of Biotechnology, Jawaharlal Nehru University, New Delhi 110067, India; jaydpb@gmail.com; 4 School of Pharmacy and Pharmacology, University of Tasmania, Hobart 7001, Australia; alok.paul@utas.edu.au; 5 School of Allied Health Sciences and World Union for Herbal Drug Discovery (WUHeDD), Walailak University, Nakhon Si Thammarat 80160, Thailand; 6 CICECO-Aveiro Institute of Materials, University of Aveiro, 3810-193 Aveiro, Portugal; mlourdespereira@ua.pt (M.d.L.P.); sonia.oliveira@uon.edu.au (S.M.R.O.); 7 Department of Medical Sciences, University of Aveiro, 3810-193 Aveiro, Portugal; 8 Hunter Medical Research Institute, New Lambton 2305, Australia; 9 Department of Microbiology, Sikkim Manipal Institute of Medical Sciences, Sikkim Manipal University, Gangtok 737102, India; kgdolma@outlook.com; 10 Department of Biotechnology & Genetic Engineering, University of Development Alternative, Lalmatia, Dhaka 1207, Bangladesh; rahamatm@hotmail.com; 11 Department of Clinical Tropical Medicine, Faculty of Tropical Medicine, Mahidol University, Bangkok 10400, Thailand

**Keywords:** hydrogels, drug delivery, therapeutic interventions, clinical trials, translation, biomedical perspectives, contact lenses, wound management, tissue engineering

## Abstract

Hydrogels are a promising and attractive option as polymeric gel networks, which have immensely fascinated researchers across the globe because of their outstanding characteristics such as elevated swellability, the permeability of oxygen at a high rate, good biocompatibility, easy loading, and drug release. Hydrogels have been extensively used for several purposes in the biomedical sector using versatile polymers of synthetic and natural origin. This review focuses on functional polymeric materials for the fabrication of hydrogels, evaluation of different parameters of biocompatibility and stability, and their application as carriers for drugs delivery, tissue engineering and other therapeutic purposes. The outcome of various studies on the use of hydrogels in different segments and how they have been appropriately altered in numerous ways to attain the desired targeted delivery of therapeutic agents is summarized. Patents and clinical trials conducted on hydrogel-based products, along with scale-up translation, are also mentioned in detail. Finally, the potential of the hydrogel in the biomedical sector is discussed, along with its further possibilities for improvement for the development of sophisticated smart hydrogels with pivotal biomedical functions.

## 1. Introduction

Over the previous decades, researchers across the globe have focused on formulating advanced systems of drug delivery that can optimize conventional medicinal agents in terms of clinical safety, efficacy, and easier administration. Delivery systems that provide drug release in a controlled manner have major advantages over traditional dosage types, such as reduced side effects and dosing frequency, along with increased patient compliance [1]. Substantial improvements have been carried out for the development of successful systems of drug delivery that are ideal to accomplish a desired pharmacokinetic profile [2]. Hydrogels are three-dimensional (3D) water-swollen crosslinked hydrophilic networks with water retaining capacity, which can withstand dissolving in water or other biological fluids [3,4,5]. Hydrogels have a high-water content that helps them in mimicking the natural tissue environment. The water absorption in hydrogels is activated by functional groups of a hydrophilic nature such as hydroxyl, amino, and carboxyl groups in the backbone of polymer chains [6,7,8]. The interaction of naturally occurring polymers and synthetic polymers delivers hydrophilic and biodegradable chemically constructed gels with more adequate mechanical strength than physically synthesized counterparts [9,10]. Various synthetic and natural polymers involved in the hydrogel formation include poly-(vinyl alcohol), poly-(2-hydroxyethyl methacrylate) (HEMA), poly-(N-isopropyl-2-crylamide), chitosan, hyaluronic acid, gelatin, and sodium alginate [11,12]. Synthetic polymers are those materials that are the outcome of a laboratory process, whereas natural polymers are derived from natural sources. Hydrogel has the capacity to absorb water in a large amount and convert it into a completely swollen state. Its absorption is almost (10 to 20) times its dry weight, therefore they are also called super absorbent hydrogels [13]. The first documented crosslinked HEMA hydrogel was formulated by Wichterle and Lim in 1960 [14]. Hydrogel synthesis is possible in numerous physical forms such as nanoparticles, films, microparticles, coating, and slabs [15,16]. The smooth and flexible characteristics of hydrogels have the advantage of minimizing inflammation in nearby tissues [17]. The porous network structure is a prerequisite for drug loading and release of bioactive agents. Various factors such as hydrogel attraction to the aqueous conditions under which they have become swollen and crosslinking agent density in the gel matrix control the porosity of the structure. Furthermore, the porous hydrogel structure enables efficient bioactive agent loading in the gel matrix and the drug release at a controlled rate, depending on the diffusion coefficient of minuscule or big molecules [14].

The classification of hydrogel network structures on the basis of pore size includes macroporous, nonporous, and microporous [18]. Macroporous hydrogels have a large pore size in the range of 0.1 to 1 μm, and the drug release from these hydrogels occurs via a diffusion coefficient mechanism. Nonporous hydrogels possess macromolecular characteristics with a size range of 10–100 Å, similar to mesh-like constructs produced as a result of monomer or polymer chain crosslinking and drug release via a diffusion coefficient mechanism [19,20,21]. In the case of the microporous hydrogel, the size of pores normally -scales between 100–1000 Å, and drug release from microporous hydrogels occurs via convection and diffusion [14,22]. The crosslinking in hydrogels is of two types comprising physical and chemical crosslinking. Numerous methods involved in physical crosslinking of hydrogels are crystallization [23], hydrogen bonding [24,25], host–guest interaction [26], hydrophobic interaction [27,28] and ionic interaction [29,30,31]. On the other hand, hydrogel formulations that are chemically crosslinked have covalent or ionic bonds among the polymer chains [32,33], and their preparation occurs by techniques such as polymerization through irradiation, [34,35,36], copolymerization [37,38], click chemistry, dynamic covalent chemistry and enzymatic crosslinking [39,40].

The biocompatibility of hydrogels has been proved from use in various areas such as an exogenic barrier in the peritoneum and as contact lenses [41,42,43]. Hydrogels provide massive social and economic benefits and reveal significant potential in the biomaterial sphere of research, application and production [44,45]. Due to the swelling properties of the hydrogel, its washing is easy for eliminating unreacted content or uncrosslinked polymers [46]. Synthetic hydrogels have progressed throughout the previous twenty years in their higher strength, extended service life, and more water imbibing ability [47]. Synthetically synthesized polymers generally possess well-defined structures that can be adjusted to produce customized functionality and degradability [48,49]. Hydrogels can be prepared from both synthetic and natural polymers; however, sometimes hydrogels can be synthesized by using synthetic polymers because they demonstrate stability under conditions of sharp and heavy temperature variations [13]. This manuscript discusses the hydrogel as a drug delivery system with its preparation methods and various polymers for fabrication aspects. Evaluation parameters are also described in addition to its drug delivery approaches for several disorders. Patents, clinical trial data, and scale-up considerations for translation on hydrogel are also stated in the current manuscript, along with potential applications in the biomedical sector.

## 2. Preparation Methodology

**Figure 1:**
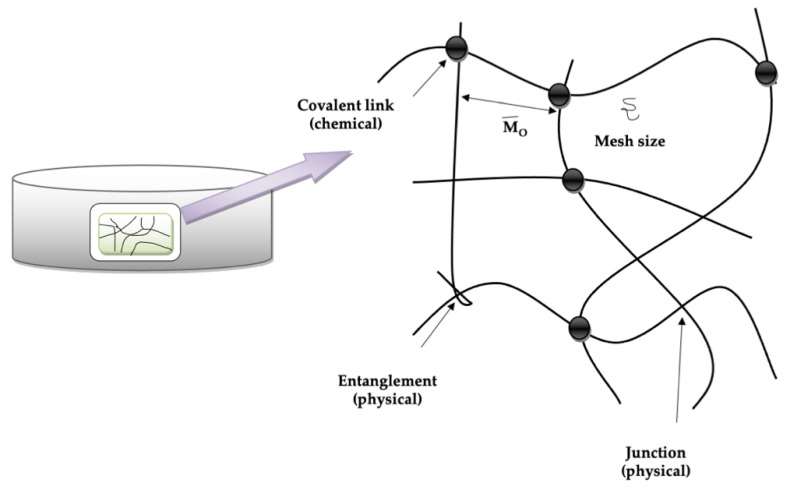
Hydrogel structural chemistry [50].

Hydrogels are three-dimensional water-swollen crosslinked hydrophilic networks with water retaining capacity and which withstand dissolving in water or other biological fluids [50] (Figure 1). Hydrogel crosslinking techniques can be divided into methods of physical and chemical crosslinking. The types of crosslinkages will determine the hydrogel characteristics physicochemically. Various methods for the preparation of hydrogels are discussed in the subsequent section.

### 2.1. Physical Crosslinking

**Figure 2:**
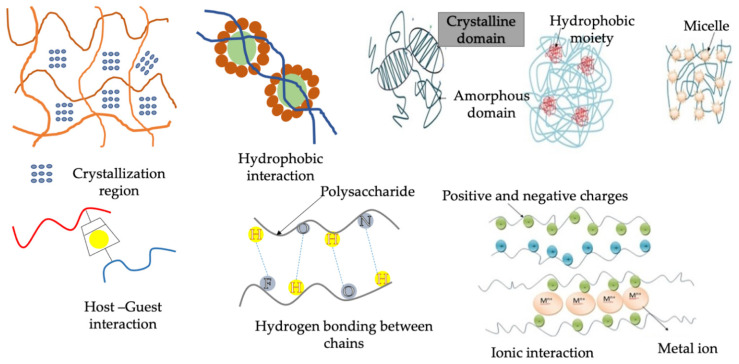
Physical crosslinking techniques.

This method involves crosslinking via physical interactions, for instance crystallization, ionic interaction, hydrophobic interaction, or hydrogen bonding (Figure 2). These techniques are achieved by freeze-thaw, cooling or heating of polymer solutions, selection of cationic and anionic polymers and pH reduction [51]. The benefits associated with crosslinking by physical means are that no supplementary chemical crosslinkers are needed for hydrogel fabrication, thereby avoiding the toxicity caused by additional crosslinkers. The semipermanent crosslinked points will help in stabilizing the 3D network and prevent the hydrogel from dissolving. For the hydrogel formation by physical crosslinking, numerous techniques have been explored.

#### 2.1.1. Hydrogen Bonding among Chains

Hydrogen bonding is a weak interaction that performs a vital role in the hydrogel preparation. In reaction to pH, solvent, or temperature change, the complex degradation and restoration of hydrogen bonds take place. The protonation of carboxylic acid groups is the reason behind the formation of hydrogen bonds [50]. Zhao et al. formulated a poly (N-vinylpyrrolidone)/gallic acid (PVP/GA) self-healable composite hydrogel using hydrogen bonding. The designed hydrogel depicted outstanding self-healing action and can be employed in body movement monitoring equipment and wearable biosensors [52].

#### 2.1.2. Ionic Interaction

The ionic interaction is a technique for hydrogel formation in which crosslinking in polymers can be carried out through the addition of di- or tri-valent counter ions. This approach is based on the theory of gelling of a polyelectrolyte solution with multivalent opposite charge ions [53]. A chitosan derivative-based hydrogel designed using ionic interactions was investigated by Yuan et al. These hydrogels were fabricated in a 2-hydroxypropyltrimethyl ammonium chloride chitosan (HACC) solution through acrylic acid (AAc) polymerization. The PAAc/HACC hydrogels demonstrated higher mechanical strength and a stronger capacity for self-healing due to the dynamic ionic interaction [54].

#### 2.1.3. Crystallization

Crystallization is another technique of physical crosslinking that is carried out using the freeze–thaw method; for instance, poly (vinyl alcohol) (PVA) hydrogels are fabricated by this method. The gel of mechanically lower strength is formed after storing aqueous PVA solutions at room temperature. Interestingly, the formation of solid elastic gel occurs when aqueous solutions of PVA go through a freezing–thawing cycle. The molecular weight and concentration of PVA, number of freezing cycles, temperature, and duration are the factors affecting the properties of the gel [55]. The composite hydrogel of curdlan (CD)/polyvinyl alcohol (PVA) was designed using a freeze–thaw process described by Ding et al. The composite hydrogel, though being remarkably ductile, had greater mechanical strength, and good cytocompatibility. As a scaffold, established porous biohydrogels could provide an optimal cell growth environment and can also be employed further in soft tissue engineering applications [56].

#### 2.1.4. Hydrophobic Interactions

Self-assembly of amphiphilic nature polymers into gel occurs in the aqueous solution through hydrophobic interactions, as a result of discordancy of hydrophobic and hydrophilic moieties thermodynamically. Hydrophobic interactions employed for hydrogel fabrication act mainly via two approaches. Firstly, micelles are utilized as a crosslinker and then micelles are loaded with drugs of hydrophobic nature while serving as crosslinking points [57]. A hydrophobically modified gelatin (HMG) hydrogel was fabricated with dissolving HMG into dimethyl sulfoxide by Takei et al. HMG hydrogel loaded with basic fibroblast growth factor stimulated neovascularization. Results revealed that as a carrier, the HMG hydrogel has considerable potential for drugs of hydrophobic and hydrophilic nature [58]. Jiang et al. prepared thermogel for melanoma using poly [(R)-3-hydroxybutyrate-(R)-3-hydroxyhexanoate] (PHBHx) functionalized with poly (ethylene glycol) and polypropylene glycol (PPG) oligomers to form PHBHx incorporated polyurethanes (PHxEP) loaded with docetaxel (DTX). DTX-incorporated thermogel exhibited an improved anti-melanoma effect. The findings suggested that the designed thermogel could be a promising carrier for long-term anticancer medication delivery [59].

#### 2.1.5. Crosslinking via Host-Guest Interactions

The host–guest interaction is a vital crosslinking technique for hydrogel production amid the numerous noncovalent interactions. Host–guest inclusion can be facilitated using numerous noncovalent interactions, such as hydrophobic interactions, hydrogen bonding, van der Waals, and electrostatics [60,61]. Moreover, interactions between the host and the guest can be reversed, with only one host and one guest. Polymers can be incorporated in a simple and reversible fashion via host–guest inclusion, facilitating the formation of hydrogels. Typically, a molecule having a larger cavity volume is called host, for instance, calixarenes (Cas), cyclodextrins (CDs), and crown ethers cucurbiturils (CBs). On the other hand, guests possessing complementary shapes can interact with the hosts [62,63]. In solutions of aqueous nature, interactions of host-guest are usually mediated through guest molecules of hydrophobic nature encapsulated in hydrophobic cavities. Among numerous host molecules, CDs are common due to their hydrophilic surface on the outside and hydrophobic cavity on the inside. For instance, PEG can help to form host–guest complexes with cyclodextrin, and it can also crosslink to form bigger aggregates or threads between a sequence of CD molecules [64].

It has been concluded that physically crosslinked hydrogels due to various advantages such as their safe and non-toxic ature are widely used in drug delivery and tissue engineering applications. However, properly addressing some issues, such as weaker bond formation and lower degrees of crosslinking, will help in designing advanced hydrogels with better efficiency.

### 2.2. Chemical Crosslinking

This type of crosslinking causes the formation of covalent bonds between polymer chains, which produces a permanent hydrogel network. Chemical crosslinking is prevalent because it can deliver better mechanical strength hydrogels [65]. Techniques mentioned in the following segment explain the synthesis of hydrogels via chemical crosslinking.

#### 2.2.1. Click Chemistry Reaction

This is a bio-conjugation type reaction in which substrates react with specific biomolecules; hence, they denote stereo-specificity of the hydrogel formation reactions. In many applications, click reactions join a biomolecule and a reporter molecule to form a product as a hydrogel. The click chemistry reaction is a Diels alder-type cycloaddition reaction, which involves the reactions between the electron-rich dienes and dienophiles. Nowadays, different types of dienes and dienophiles with furan rings are used to provide a range of hydrogel formations. These types of reactions have advantages of providing products without the undesired side product formation [66].

#### 2.2.2. Schiff Base Approaches

The Schiff base is the imine linkage intermediates formed from the reactions between aldehydes and amino groups. The Schiff bases are utilized in the hydrogel formation through the uncoupling and recoupling imine linkages, resulting in advantageous self-healing property. The polymeric hydrogel formed by the self-crosslinking reaction between alginate dialdehyde groups (ADA) and the acrylamide-modified chitin amino groups (AMC) depicts that the self-healing property depends on the concentration of the substrate molar ratios and the neighboring pH [67]. The novel injectable hydrogel is formed by the Schiff bases reactions between an aldehyde and hydrazine functional moieties, for example, hyaluronic acid (HA-CHO) as aldehyde and hydrazide-modified poly (γ-glutamic acid) (γ-PGA-ADH). These types of hydrogels have advantages of mechanical and biocompatibility properties [68].

#### 2.2.3. Oxime Crosslink Method

The oxime is formed through a chemical reaction with an aminooxy type group, such as hydroxylamine functional groups, with different types of aldehyde or ketone moieties. This reaction is chemoselective, reacts selectively, and can be performed in the presence of various functional groups of substrates. This oxime bond formed shows enhanced hydrolytic stability and is more stable than the hydrazine bonds [69]. There are reports that the self-healing property of the oxime-functional hydrogels shows changeable gel-to-sol transition through the oxime functional group interchanged with other groups, and the reaction requires acidic conditions. The synthesis of oxime hydrogel is performed between the keto-functional P (DMA-stat-AA) and difunctional alkoxyamines with the property of reversible gel-to-sol transition polymer solutions [70].

#### 2.2.4. Michael Type Addition Reaction

Michael addition is a reaction involving nucleophiles (which act as a donor) and activated electrophiles such as olefins or alkynes (which act as an acceptor), which form new carbon-carbon bonds. In the hydrogel formation, the Michael addition type reaction is performed between the thiol functional group-containing polymers and activated α,β-unsaturated carbonyl polymers in the basic medium. The chitosan-based hydrogel formed from the Michael addition type reaction, in which the thiolated chitosan polymers react with the poly (ethylene glycol) diacrylate (PEGDA), show higher mechanical strength. The reaction between the dithiothreitol (DTT) and glycidyl methacrylate derivatized dextran (Dex-GMA) in the presence of phosphate buffer is an example of Michael addition type hydrogel [71,72].

#### 2.2.5. Enzymatic Crosslinking

Enzymatic crosslinking is an efficient technique for hydrogel fabrication in the presence of mild conditions and is better for avoiding unintended side reactions due to organic solvents or photo-initiators. However, it is costly, and the specificity of the substrate restricts its application. An acyl-transfer reaction between the glutamine residue group γ-carbonyl and the lysine residue group ε-amino is catalyzed by microbial transglutaminase [73,74,75]. Enzymatically crosslinked hydrogels comprising silk fibroin SF substituted with tyramine SF- SF-TA or gelatin G-TA were investigated by Hasturk et al. Both G-TA and SF-TA substantially enhanced gelation kinetics, increased tensile strength, and prolonged enzymatic degradation. These results clearly indicate that biocompatible hydrogel formulations can be employed in cell delivery and tissue engineering [76].

#### 2.2.6. Radiation Crosslinking

Radiation is a safe alternative for hydrogel fabrication, conducted using high-energy radiations such as γ-irradiation and ultraviolet (UV) light irradiation. Conversion of inert groups to active groups or unsaturated bond polymerization may be initiated due to the production of radicals on polymer chains after radiation and exposure. This method is free from additives and sterilization, and gel formation occurs concurrently [77]. A radiation crosslinking technique was employed to fabricate carboxymethyl hyaluronic acid (CMHA) hydrogels by Relleve et al. CMHA hydrogels demonstrated good swelling and biocompatibility, which might open an extensive application range of hydrogels processed by radiation [78].

#### 2.2.7. Hydrogel Crosslinking by Dynamic Covalent Chemistry

The use of a dynamic covalent chemistry approach for crosslinking is particularly fascinating since it helps in hydrogel formation with the same viscoelastic properties as those found in human tissues. Dynamic covalent bonds are chemical bonds of reversible nature that can reform and split into time scales, relevant for cell-based matrix remodeling. Products that are in a state of dynamic equilibrium with their reactants are included in the category of dynamic covalent chemical bonds. Boronic ester, for example, is a compound with dynamic covalent connections that offers a lot of potential for producing self-healing hydrogels. Materials formed using covalent links are more stable than physical networks, and dynamic covalent bonding is capable of producing a material with mechanical properties of the physical network, which imitates the features of soft tissues in a better manner. Dynamic covalent chemistry can be used as an auxiliary to create viscoelastic materials that relax and deform in the presence of stress to help cellular activities such as spreading, proliferation, and differentiation [79].

From these research observations, it is evident that chemically crosslinked hydrogels are known for their increased stability under physiological settings, good mechanical qualities, and tuneable degradation behavior. However, comprehensive research efforts should be made to minimize the cellular toxicity of chemical crosslinkers in order to design hydrogels for wider biomedical applications.

## 3. Polymers Employed in Hydrogel Fabrication

Novel delivery techniques have been developed as a result of advances in polymer science. The development of polymers with unique properties has occurred from the introduction of novel polymers. Polymers are crucial in the evolution of drug delivery technology over long periods because of their potential to provide therapeutic agents in a controlled manner along with the tunable release of both hydrophilic and hydrophobic drugs [80,81,82]. Widely used polymers in hydrogel fabrication are mentioned in the following section.

### 3.1. Polyvinylpyrrolidone

This is a polymer with aqueous solubility produced from N-vinylpyrrolidone monomer through a process of radical polymerization. It is also named povidone or polyvidone. Polyvinylpyrrolidine (PVP) is nontoxic, biocompatible, and has a complex affinity for both hydrophilic and hydrophobic drugs [83,84,85]. PVP is employed widely in hydrogel formulations. Grafting of crotonic acid (CrA) with PVP resulted in a pH-responsive hydrogel that showed low release in acidic pH and better-prolonged release in neutral pH, indicating the feasibility of using this hydrogel as a potential drug carrier for targeted release in the intestinal medium [86]. Highly elastic and superabsorbent hydrogels fabricated with collagen-(PVP)-poly (acrylic acid) (PAA)-poly (ethylene oxide) (PEO) demonstrated superabsorbent ability in simulated biological buffers and deionized water. The hydrogel was maintained in conditions simulating both the pH of the healthy skin and an infected wound for more than 50 h [87].

### 3.2. Polyethylene Glycol

Polyethylene glycol (PEG) is a polyester of synthetic origin, soluble in water. Due to the biodegradable and biocompatible nature of PEG, it has been employed more frequently for hydrogel preparation. Hydrogels formed using PEG have shown huge biomedical potential as matrices for controlled release of therapeutics or as scaffolds for facilitating the regeneration of tissues [88]. For prolonged action of celecoxib in joints, hydrogels have been designed using poly (caprolactone-co-lactide) (PCLA)-PEG-PCLA triblock copolymers by Petit et al. Findings demonstrated that the hybrid crosslinked hydrogel exhibited slower release in addition to lessened burst release of celecoxib, and this drug was found in the synovial fluid for two months. It can be concluded from findings that this newly designed hydrogel system displayed substantial promise as a platform for further progress in delivery via intra-articular route [89]. PEG hydrogel was designed by encapsulating collagen into it via the photopolymerization method. The formed hydrogel demonstrated an understanding of cell motility dynamics [90]. Hydrogel formulated using chitosan and maleic terminated polyethylene glycol (PEG-MA) displayed a porous structure with an excellent swelling ratio and biocompatibility. Hydrogel depicted faster-wound closure, and, therefore, has the potential to heal wounds [91].

### 3.3. Polyvinyl Alcohol

This is a synthetic polymeric material with high aqueous solubility and is biologically degradable. This polymer can be prepared by the hydrolysis of polyvinyl acetate and is among the most important class of synthetic polymers used in nutraceutical and medical applications [92]. Polyvinyl alcohol (PVA) is a widely used polymeric material in hydrogel formation that has use in drug delivery, cell encapsulation, and wound dressings. PVA/citric acid (CA)/Ag nanoparticles (NPs) fabricated hydrogel loaded with ciprofloxacin were developed by Sabzi et al. Hydrogels prolonged ciprofloxacin release, and PVA matrix incorporated with ciprofloxacin, Ag NPs, and CA delivered an effectual antibacterial action towards microorganisms such as *Escherichia coli* and *Staphylococcus aureus*. For protracted antibiotic treatment, it has been revealed that formulated hydrogels can prove to be a beneficial material [93].

### 3.4. Poly (Acrylic Acid)

This is a synthetic anionic polymer of acrylic acid [94]. Hydrogels of poly (acrylic acid) (PAA) and polymethacrylic acid (PMA) for insulin delivery in a controlled manner were formulated by Mallawarachchi et al. Results stated that PAA hydrogels are beneficial as a delivery system for regulated drug release [95]. A nanocomposite hydrogel of polymers gelatin (Ge)-g-poly (acrylic acid-co-acrylamide) and montmorillonite (MMT)-clay- with vitamin B12 showed controlled release of vitamin B12 in artificial gastric fluid (AGF) and artificial intestinal fluid (AIF). In AGF, the release was 42%, and in AIF, the total release was 80% over 6 h [96].

### 3.5. Poly-N-Isopropyl-Acrylamide

A thermoresponsive polymeric material which has been extremely explored for hydrogel formulation is poly (N-isopropyl-acrylamide) (PNIPAM), considered as a potential candidate for biomedical applications [97]. Well-designed poly(N-isopropyl-acrylamide)/mesoporous silica nanoparticles (MSN) composite hydrogels reveal that the addition of MSN leads to improved network structure with enhanced drug loading capability. Composite hydrogels demonstrated temperature-activated release of drugs and enormous potential for drug transportation [98]. Fabricated injectable hydrogels of poly (N-isopropyl-acrylamide) and carboxymethyl chitosan, i.e., poly (CMCS-g-NIPAAm) encapsulated with 5-fluorouracil, displayed cytocompatibility and maximum release in acidic pH and regulated cytotoxic capacity in depot form. Observations inferred that the prepared formulation has the potential of intratumoral and systemic controlled drug delivery properties [99].

### 3.6. Polyurethane

Polyurethane (PU) is a synthetically produced polymer, comprising organic units joined by carbamate links. Hydrogels fabricated by polyurethane and chitosan for the release of 5-fluoro uracil demonstrated controlled drug delivery [100]. PU hydrogels showed excellent self-healing capability, higher mechanical strength, and greater stretching toughness [101]. Chitosan-polyurethane hydrogel membrane (HPUC), designed by Viezzer et al., showed minimal cytotoxicity and better healing action on wounds when employed with mononuclear bone marrow fraction cells. Results indicated that the HPUC is an ideal candidate for wound healing [102].

### 3.7. Hyaluronic Acid

Hyaluronic acid (HA), also named hyaluronan, is an anionic, nonsulfated glycosaminoglycan present in various parts of eyes, skin, and connective tissues [103]. It mainly functions by retaining water for keeping our tissues moist and well lubricated. A paclitaxel (PLX)-loaded hydrogel was developed using HA and cationized reduced graphene oxide (rGO) sheets by Patil et al. The formulation displayed offered sustained release of PLX and HA resistance was elevated significantly with sheet incorporation, and it showed improved biocompatibility of cationized rGO [104]. The thermo-sensitive in situ gelling formulation based on ketoconazole (KCL) and poly (N-isopropylacrylamide)/hyaluronic acid was employed in the fabrication of the hydrogel. Experimental outcomes stated that KCL release from gels was moderate and without burst effects in addition to preventing *Candida albicans* growth. Hence, from the results, it can be concluded that this newly designed hydrogel formulation for the eye could prolong the duration of residence and control the release of the drug [105].

### 3.8. Chitosan

This is a polymer containing randomly disseminated β-linked D-glucosamine and N-acetyl-D-glucosamine units. It has numerous applications in the biomedical sector because of its inertness, biocompatibility, degradability, and ease of chemical modification [106]. Montmorillonite-loaded pH-responsive and magnetic κ-carrageenan/chitosan hydrogels were fabricated by Jafari et al. for determining the release of sunitinib as a drug. The designed hydrogels were prepared via ionic crosslinking in the presence of magnetic montmorillonite (mMMt) nanoplatelets. Results depicted that mMMt addition resulted in a change in the microstructure of hydrogels along with elevated drug loading efficiency of nanocomposite hydrogels. The in vitro release data showed sustained release of sunitinib. The fabricated hydrogel showed a higher capacity for loading of a drug, and subsequent pH-sensitive drug release could be used in protracted cancer treatment with fewer adverse effects. Hence, it can be concluded that the designed hydrogel has enormous potential in drug delivery as a carrier [107]. Adalimumab-loaded hydrogel eye drops comprising β-glycerophosphate and low-deacetylated chitosan have been fabricated. Results demonstrated that adalimumab-incorporated hydrogel eye drops were highly effective in comparison to the free drug both in clinical efficiency and permeation rate. This strategy is considered clinically beneficial for ophthalmic medication [108].

### 3.9. Gelatin

This is a highly biocompatible natural polymer obtained from collagen hydrolysis [109]. Formulated gelatin hydrogel conjugated with 5-aminopyrazole (5-AP/G) and loaded with the drug 5-fluorouracil (5-FU) demonstrated drug release in a predictable manner under rectal conditions with remarkable cytotoxicity. Results suggested that hydrogel for rectal administration of 5-FU could be employed in the future for rectal cancer therapy [110]. Gelatin (Gel)/PVA hydrogels were loaded with methotrexate (MTX) for colon targeted delivery. These findings reveal that the produced Gel/PVA hydrogels can be potential carriers for MTX delivery to the colon in a controlled manner [111].

### 3.10. Sodium Alginate

Sodium alginate (SA), a natural polymeric material comprising residues of 1,4-linked-β-D-mannuronic acid and α-L-guluronic acid, has exceptional biocompatibility, immunogenicity, non-toxicity, and biodegradability, making it an outstanding polymer for drug delivery [112]. Hydrogels formulated using SA are also beneficial for the release of drugs and biologically active molecules in a controlled manner. The hydrogel of SA/polyvinyl alcohol in combination, encapsulated with rosuvastatin-loaded chitosan (CS) nanoparticles, has been fabricated. Observations showed that SA:PVA ratio of 7:3 and 3 wt % of drug-loaded CS nanoparticles resulted in optimal mechanical strength of hydrogel film, and the entire loaded drug was released within 24 h [113]. Various studies reported in the literature confirm that the designed hydrogels, owing to their inherent properties, could be utilized as a potential carrier for delivery of various therapeutic agents safely and effectively.

The last few decades have been a boon for research innovation in polymer therapeutics, as it facilitated innocuous and efficacious delivery of bioactive agents for treating a wide range of medical disorders. Biodegradable and bio-reducible polymers are an admirable and outstanding option for hydrogel fabrication. In the future, combining synthetic and natural polymers will deliver a new paradigm for producing an effective hydrogel drug delivery system.

## 4. Evaluation Parameters

**Figure 3:**
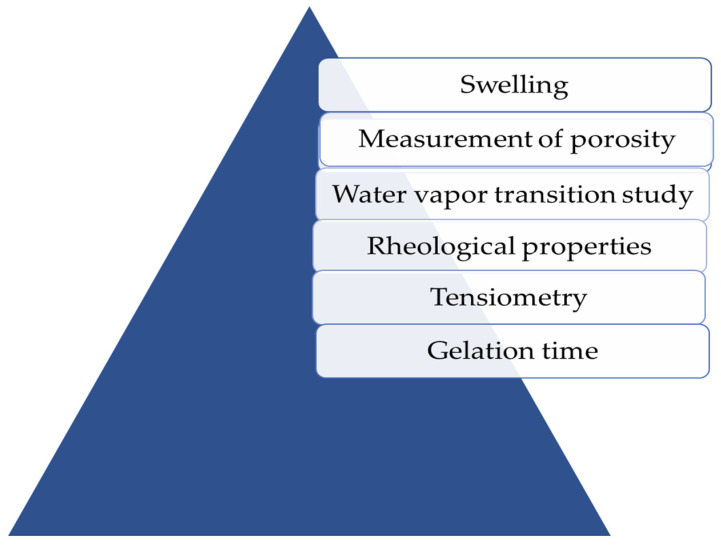
Evaluation parameters of hydrogels.

Evaluation is an extensively considered parameter after formulation, and there are various techniques for hydrogel evaluation (Figure 3). Some popular techniques utilized in the evaluation aspects are portrayed in the following section.

### 4.1. Gelation Time

Gelation time relates to the amount of time it takes for a polymer formulation in the liquid form to transform into a gel. The hydrogel exhibits in situ gelation phenomena through ionic crosslinking or after temperature or pH alteration. Determination of a sol-gel transition period via a technique of inverted tube is well known [114]. For this analysis, tubes holding solutions of discrete concentrations in final form can be equilibrated at 37 °C, and at varying time periods while the tubes are inverted. The duration during which the flow was not seen in gel is considered as the time of gelation [115]. The sol-gel phase transition behavior of hydrogels can be also investigated by rheometry. In order to obtain sophisticated hydrogels with desired mechanical, physicochemical and functional attributes, the study of sol-gel transition with sensitive rheological method is also needed.

### 4.2. Porosity

Porosity is a significant parameter in hydrogel evaluation. Here, for measuring the porosity, the technique of solvent replacement is well recognized by the scientific community. Pores in the hydrogel matrix help in loading drugs in huge amounts and simultaneously aid in increasing the release rate of drugs. This method starts with weighing the hydrogel discs, before placing them entirely in pure ethanol. Discs are separated from the ethanol after an approximate duration and dabbed by means of blotting paper for extracting excess ethanol lying on the disc surface, then again weighed. The equation for % porosity calculation is given as

$$Porosity\;\left(\%\right)=\frac{W_{2}-W_{1}\;}{\rho V}\times 100$$

where *W*_2_ and *W*_1_ represent the weight of hydrogel discs after placing in absolute ethanol, respectively, ρ is ethanol density, and *V* is hydrogel volume [116].

### 4.3. Swelling

Swelling is an ongoing transformation phase from an unsolvated glass or partially rubbery condition to a relaxed rubbery zone. Through this analysis, water absorption in the hydrogel is measured by weighing the samples initially, followed by putting them in 5 mL phosphate-buffered saline (PBS) solution (pH 7.4) at 37 °C for a duration of 24 h and, finally, samples are dried. The absorption of water is calculated based on the following equation [114,117].

$$Swelling\;\left(\%\right)=\frac{M_{s}-M_{d}\;}{M_{d}}\times 100$$

where *M_s_* and *M_d_* represent the mass of swollen and dried discs, respectively.

### 4.4. Water Vapor Transition Rate

The water vapor transition rate (WVTR) is also an important hydrogel evaluation method that indicates the permeability of a hydrogel sample to water vapor. The hydrogel intended for the healing of wounds should not exhibit high or low values of WVTR; the wound would get dehydrated or inflamed. The analysis is carried out by cutting hydrogel film individually in the shape of a square with a thickness of 3 mm and a diameter of 40 mm. Subsequently, the film is mounted on the Falcone pipe as a cap having a diameter of 35 mm and a pipe containing distilled water of 25 mL volume. The entire device can be transferred into a fully isolated chamber, with stable humidity and temperature (35% and 35 °C) for one day [118,119]. The WVTR is calculated using the following equation:

$$Water\;vapor\;transition\;rate=\frac{\Delta W\;}{\Delta T\;\times \;A}\;{\mathrm{mgh}}^{-1}{\mathrm{cm}}^{-2}$$

where Δ*W*/Δ*T* is the amount of water gain per unit time of moisture transfer, and *A* is the area exposed to the water surface in cm^2^.

### 4.5. Rheological Properties

Rheological evaluation of hydrogel delivers significant facts about distortion in materials when subjected to external forces. Rheological evaluation is generally carried out employing a rheometer having a gap among plates usually of 1 mm in parallel plate geometry (diameter 25 mm) cell. However, rheometer plate gap may vary depending on characteristics of a particular material. Other geometrical plates (cone plates) can also be employed for rheological characterization of hydrogel. Hydrogel is sliced into a cylindrical form, the elastic modulus (G’) and viscous module (G”) is recorded at a constant strain of 1 percent and 0.5 percent over a frequency range of 0.1–100 rad/s, for which they are in the linear viscosity range [120,121].

### 4.6. Tensiometry or Tensile Tests

This test is employed for calculating numerous hydrogel properties using tensile force, for instance, the young’s modulus, tensile strength, and yield strength. The tensile force is applied amid two grips of the material, which can then be obtained by measuring the applied force and hydrogel elongation/elasticity from the stress-strain graph. For evaluating viscoelastic hydrogel properties, the stress relaxation and material elongation parameters are utilized [122]. The mechanical properties of hydrogels are determined by using the compression test. This test involves putting hydrogel material between the plates and applying pressure on the surface of the hydrogel by compressing the plates. It is then possible to measure the mechanical properties theoretically, and the compression distance along with the pressure is also employed to measure the properties [123].

It was concluded that the aforementioned evaluation parameters play vital roles in estimating the performance of hydrogel. Evaluation parameters have a significant impact on a product’s processability and final attributes.

## 5. Hydrogel Drug Delivery Systems in Various Disorders

Hydrogels act as a promising carrier for the delivery of drugs as they protect various bioactive agents from hostile conditions of the body and therefore have attained great interest of researchers (Table 1). 

**Figure 4:**
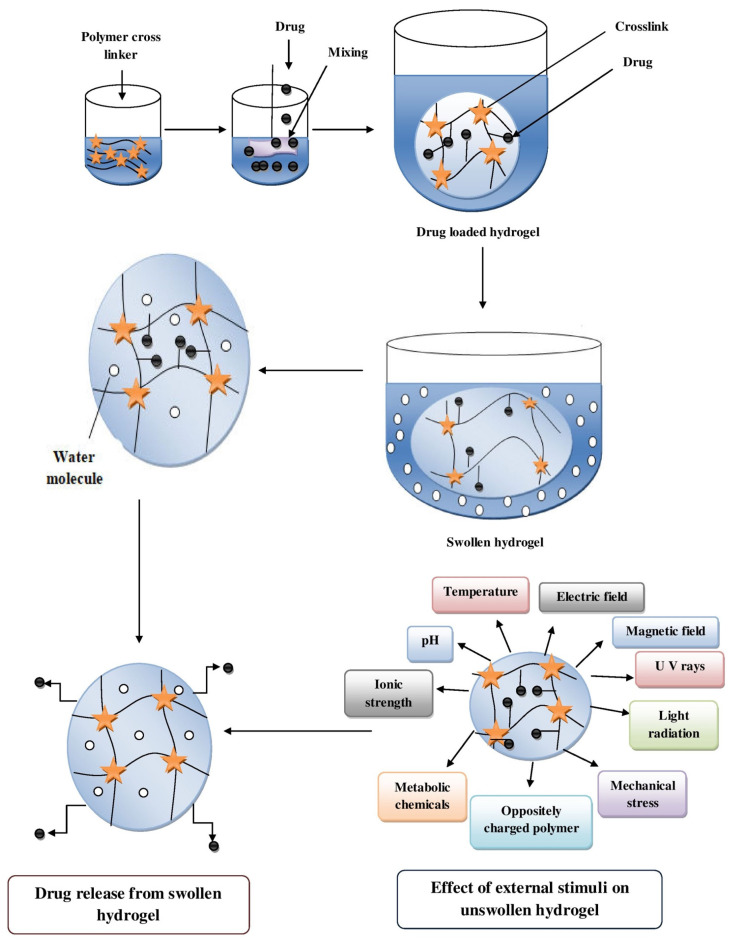
Mechanisms of drug release from hydrogel.

The most common method of releasing the drug or molecules from the hydrogels is passive diffusion [129,130]. In terms of the release behavior of therapeutic agents, the hydrophilic nature of a hydrogel distinguishes it from non-hydrophilic polymer matrices. Drug release mechanisms from hydrogels can be classified as diffusion controlled, swelling controlled and chemically controlled. Diffusion-controlled behavior is the most widely applicable mechanism to characterize the drug release from hydrogels. The mesh size of a hydrogel matrix influences the diffusion of drug out of the matrix of the gel. The degree of crosslinking, chemical structure of components, type, and intensity of the external stimuli also alter the drug diffusion out of a hydrogel matrix. If diffusion of a drug is substantially faster than the distention of a hydrogel, the swelling-controlled mechanism plays an important role in release behavior study. Furthermore, chemical reactions occurring within the gel matrix dictate chemically controlled release. There are three important functions of hydrogels, i.e., controlled release rate, drug storage region, and drug release drive, which maintain the effective release of the entrapped drug into the body after administration. With this, by altering the gel structure with different stimuli i.e., temperature, electric field, pH, and ionic strength, the release of a drug can also be controlled easily (Figure 4). Though the delivery of a lipophilic drug through hydrogel is difficult due to non-uniformity, poor solubility, limited in vivo stability, and dissolution problems, it can be improved to some extent by enhancing solubility, stability, and bioactivity so that the release of a drug can occur in a controlled or sustained manner [41,130]. Moreover, hydrogels can also mask the bitter taste and odor of medications, and no systemic toxicity is seen in hydrogel-based delivery systems [41,130]. Hydrogel drug delivery approaches for various disorders are discussed in the subsequent section.

### 5.1. Hydrogel for Conjunctivitis

Conjunctivitis is an eye disorder that causes conjunctival vessel dilation that results in edema and hyperemia of the conjunctiva, usually with associated discharge. Conjunctivitis affects a large segment of the population of United States annually [131]. A hydrogel formulated using chitosan and poloxamer 407 with model drugs (i.e., neomycin sulfate and betamethasone sodium phosphate) demonstrated no eye irritation in rabbits. It was revealed from various experimental findings that a prepared hydrogel formulation can be employed as a substitute to traditional eye drops for providing extended therapy for conjunctivitis treatment [132]. DNA/poly (lactic-co-glycolic acid) (PLGA) hybrid hydrogel (HDNA) loaded dexamethasone (DEX) was designed for allergic conjunctivitis. These hybrid HDNA hydrogels showed biodegradability and biocompatibility along with significantly increased DEX retention. Hydrogel mediated the progressive DEX release in eye cells and tissues. Therefore, HDNA-based ophthalmic treatment methods enable new paradigms to treat different eye disorders [133].

### 5.2. Hydrogel for Psoriasis

A common skin problem is psoriasis, which affects around 3% of people worldwide [134]. A crosslinked interpenetrating polymer network (IPN) hydrogel of luteolin drug was fabricated using hyaluronic acid (HA) and poly (N-isopropylacrylamide) (PNIPAM). HA/PNIPAM IPN hydrogel provided efficient luteolin delivery to the dermis and epidermis, and no toxicity was seen in the results. It can be concluded from the findings that IPN hydrogels can be fabricated for transdermal delivery of luteolin for skin relief in psoriasis [135]. The prepared tacrolimus (TAC)-loaded composite hydrogel for psoriasis lesions depicted local tolerance with no immediate toxicity signs after repetitive administration in mice topically with substantial enhancement in the in vivo features. It has been found from evaluation results through the imiquimod-induced psoriasis model that skin delivery of TAC hydrogel composite is twice as strong as its commercial formulations [136].

### 5.3. Hydrogel for Rheumatoid Arthritis

Rheumatoid arthritis is an autoimmune disorder responsible for joint swelling and cartilage damage [137]. Betamethasone encapsulated hydrogels designed using tyramine modified gellan gum conjugated with silk fibroin (Ty-GG/SF) for treating rheumatoid arthritis showed better resistance against enzymatic degradation and liberated betamethasone in a controlled manner. Study outcomes disclosed that betamethasone-encapsulated Ty-GG/SF hydrogels are better therapeutically than the drug alone. Hence, this approach is beneficial in terms of therapeutic effectiveness compared to conventional drug usage in rheumatoid arthritis therapy [138]. A methotrexate (MTX) and indomethacin (IND) loaded temperature-sensitive hydrogel (D-NGel) incorporated with nanoparticles (D-NPs) for arthritis was developed by Yin et al. and in vivo activity was carried out on collagen-induced arthritis rats. Prepared D-NGel slowly released drugs for up to 72 h in the joint fluid. D-NGel successfully decreased swelling in joints and bone deterioration. Findings showed D-NGel potential for prolonged co-delivery of IND and MTX for synergetic rheumatoid arthritis treatment, addressing symptoms of rheumatoid arthritis and its root causes [139].

### 5.4. Hydrogel for Breast Cancer

Breast cancer is a highly prevalent form of cancer that is the second largest cause of mortality [140]. Nanocomposite hydrogel loaded with capecitabine was formulated by Taleblou and co-workers using polyvinyl alcohol and montmorillonite. Outcomes confirmed augmented anti-cancer action of the capecitabine-incorporated nanocomposite hydrogel. In vivo evaluation results on BALB/c mice showed that nanocomposite hydrogel significantly reduced tumor growth and improved effectiveness against cancer cells. Hence, the formulation can be employed to release the anti-cancer drugs in a controlled manner with greater therapeutic action [141]. A hydrogel nanocarrier was designed for injecting at the tumor site of BALB/c mice through the co-assembly of tailor-made hexapeptide and doxorubicin. The outcomes clearly indicated that hydrogel prolonged the release of the drug, which eventually led to a decrease in cancer reoccurrence. Therefore, this targeted chemotherapy technique denotes a potential adjuvant therapeutic approach for the recurrence of breast cancer [142].

### 5.5. Hydrogel for Alzheimer

Alzheimer’s disease (AD) is a debilitating brain disorder with a higher mortality rate among older adults with no effective treatment [143,144]. Hydrogel formulations for AD were formed using thiolated chitosan with liposomal donepezil HCl (LDH) and evaluated on rabbits. The mean brain content of the drug was augmented by liposomal hydrogel compared to oral DH tablets. Another study indicated that the formed formulation is efficient for delivery of DH via nasal route and could be employed for AD treatment [145]. Fabricated in situ hydrogels (ISG) loaded with timosaponin BII were formulated by which inducible nitric oxide synthase gets reduced in the brain, and tested on C57BL/6J mice. Hence, the formulated hydrogel can be a better alternative for preventing AD [146].

### 5.6. Hydrogel for Diabetes

Diabetes has been a rapidly increasing global disorder over the last three decades [147]. Hydrogel-based systems are now being investigated in diabetes in the form of injectable hydrogel. These hydrogels loaded with lixisenatide (Lixi) for type 2 diabetes mellitus (T2DM) using poly (ε-caprolactone-co-glycolic acid)-poly (ethylene glycol)-poly (ε-caprolactone-co-glycolic acid) and poly (D,L-lactic acid-glycolic acid)-poly (ethylene glycol)-poly (D,L-lactic acid-co-glycolic acid) were evaluated on diabetic db/db mice. The combined hydrogel displayed prolonged Lixi drug release, and after three consecutive administrations, it escalated the level of plasma insulin besides reduced glycosylated hemoglobin. Outcomes indicated that the Lixi loaded combined hydrogel is a promising approach for T2DM treatment [148], and the chitosan/dialdehyde starch derivatives (CS/SB-DAS-VPBA) with zwitterionic dialdehyde starch-based micelles (SB-DAS-VPBA) were produced. The micelle-hydrogel loaded with insulin and nattokinase was fabricated by Wen et al. In vitro results showed that the micelle-hydrogel delivered insulin properly and offered a strong thrombolytic action. Therefore, the formed system can also be used as a forum for treating complications of diabetes [149].

### 5.7. Hydrogel for Fungal Infections

Superficial fungal infections have increased worldwide and affect numerous body parts such as the skin, vagina, hair, buccal cavity, and nails [150,151]. A hydrogel system was designed for the anti-fungal drug ketoconazole (KZ), comprising solid lipid nanoparticles (SLNs) as well as SLN-containing hydrogel (KZ-SLN-H) for oral and topical KZ delivery on male albino Wistar rats. Findings showed drug release in a sustained manner besides improved permeability. Outcomes clearly stated that SLN and SLN-H formulations might also be viewed as effective vehicles for the delivery of KZ orally and topically to ameliorate control over systemic and topical fungal infections [152]. A luliconazole nanocrystal (LNC) integrated hydrogel formulation was prepared for fungal infections by Kumar et al. Findings demonstrated that drug retention from LNC hydrogel (N-GEL) was highest and highly efficient in fungus eradication. Thus, this novel approach of incorporating LNC with hydrogel resulted in enhanced action and improved dermal intake of drugs with low water solubility [153]. Nystatin nano-capsular hydrogel was fabricated with nanoprecipitation technique using polycaprolactone, squalene, and span 60, the developed formulation was tested on male albino rats. The formulated hydrogel showed higher encapsulation efficiency in comparison to the marketed formulation with a substantial lessening of the fungal count and candidal infection abolition. It is evident from findings that nanocapsular hydrogel is an efficient approach in treating topical candidiasis [154].

### 5.8. Hydrogel for Trichomonas Vaginalis Infections

*Trichomonas vaginalis* is a global, curable non-viral sexually transmitted infection [155]. A thermoresponsive hydrogel has been fabricated with auranofin (AF)-loaded nanoparticles (NP) for intravaginal administration. AF-NP composite hydrogel displayed drug release in a sustained manner besides outstanding NP retention with increased levels of AF locally in mice. AF-NP gel delivered intravaginally outshines oral AF in eradicating trichomonad infection with no local or systemic toxicity. Thus, results show the potential of this hydrogel formulation for vaginal infections topically [156]. Indole-3-carbinol (I3C) hydrogel comprising I3 C-loaded nanocapsules was fabricated for trichomoniasis treatment. Nanocapsules of Eudragit^®^ RS100 and rosehip oil containing I3C (NC-I3C) were formulated via interfacial deposition of preformed polymer technique. A hydrogel formulation for vaginal administration was designed (HG-NC-I3C) via thickening the NC-I3C with gellan gum. Results illustrated these formulations were non-irritating and promoted the release of I3C in a controlled manner. The designed HG-NC-I3C therefore emerged as a potential treatment strategy for trichomoniasis by vaginal administration [157].

### 5.9. Hydrogel for Tuberculosis

Tuberculosis (TB) is a chronic disease that primarily affects the lungs but can spread to various body organs such as the kidney, brain, spine, or intestine [158]. For transdermal delivery of drugs such as rifampicin, ethambutol, isoniazid, and pyrazinamide, hydrogel-forming microneedle (MN) arrays were formulated. Findings of solute diffusion revealed that the physiochemical characteristics and functional groups of each drug influenced drug penetration across the swollen hydrogel membrane. Outcomes also displayed the flexibility of hydrogel formulations to provide MN arrays with a TB drug regimen. Therefore, this can be a promising approach for supplying large doses of TB medicines [159]. Hydrogels are also performing admirably well in administering first- and second-line anti-tubercular medication regimens.

Research investigations reported in the literature confirmed that hydrogel strategies can play a pivotal role in managing several disorders. Hydrogels with customizable structural and functional features attained through modifying methods of synthesis have a lot of potential as nanocarriers for achieving site-specific drug delivery. In recent years, hydrogels demonstrated their potential for developing future tailored nanomedicine to treat infectious disorders. Despite the fact that hydrogels have been successfully used to treat various diseases, major efforts should be made towards designing and preparation of smart hydrogels in the nano-domain with encouraging features and simplicity of processing in order to produce therapies for target diseases. Furthermore, dynamic hydrogels are paving the way for new translational possibilities, and good progress is also being made in bringing biomaterials to new and acceptable level of promising hydrogel technologies.

## 6. Recent Patents, Clinical Trials and Scale-Up Considerations for Translation

The increasing number of patents and clinical trials on hydrogels show their popularity among researchers, and marketed formulations also suggest their success on the commercial scale [160]. Patent data are considered a vital information platform for the discovery of development in technical growth and huge prospects (Table 2). 

Patent analysis is an extensively employed tool for evaluation of current technological advances besides providing prediction of emergent technologies [171,172]. Technology execution is also clinically essential for validating concepts and preclinical experiments carried out in animal models [173]. Clinical investigation not only provides detailed information of treatment results but also demonstrates key details such as a patient’s health and the related therapy levels and risks of a specific treatment [174]. In order to discuss the results in a more practical and precise manner, clinicians and scientists now apply mathematical formulas, statistical techniques, and resource expenditure evaluation [175]. The clinical studies carried out so far portray enticing results in the field of hydrogels and could encourage scientists to perform research on modern approaches (Table 3). 

Overall, in order to evaluate the clinical effectiveness of various new therapeutic options and to convert the technology from lab to market, further research is also needed. Hydrogel scale-up and production for commercialization is still a challenging process. Despite the critical nature of scalable production in bringing hydrogel technologies into the clinic, this topic is less addressed in the literature. The reason behind this might be an overall absence of interest in these topics, limited interaction among academic and industry partners, or insufficiency of research finance may make it difficult for academic organizations to investigate scaled-up production. All of these factors more than likely contribute to the paucity of research into improving the process engineering of these biomaterials [193]. Various manufacturing and regulatory aspects that must be considered while fabricating hydrogels with translation in mind are briefly outlined here. On the basis of the application, hydrogels are considered as a drug, device, or biologic by the United States FDA. The fastest and most budget regulatory path would be a device having 510 (k) designation [194]. Devices get faster approval (about 5 years), but if the therapeutic agents or cells are delivered by hydrogel, it is generally categorized as a combination product that requires an approval timespan of 7–10 years in addition to USD 50–300 million for production and analysis [195]. Mainly, hydrogel fabrication is carried out in smaller batches for preclinical testing, but the production at a larger scale and processes ought to be designed as well as optimized through good manufacturing processes (GMP) before authorization and commercialization [196].

Hydrogel manufacturing must be conducted safely on a kiloton scale for widespread use. Even when going from small to large animal preclinical investigations, the significant issue of scaling up should not be overlooked. Scaling and manufacturing procedures may be affected by the chemical components of the hydrogel. If chemical moieties in hydrogels degrade over time as a result of processes such as hydrolysis, correct storage and formulation processes must be anticipated and accounted for. Individual hydrogel components may provide specific regulatory issues, particularly as biologicals and nanotechnology become more integrated into next-generation formulations. Although the benefits of incorporating nanoparticles in hydrogel formulations are obviously noticeable in the preclinical data, nanomedicines have historically been challenging for translating into the clinic [197]. Furthermore, most preclinical hydrogels are designed using biopolymers such as collagen, alginate, or cellulose, but these biopolymers frequently display batch-to-batch variance, which can make it difficult to meet stringent quality control measures. Apart from generating individual hydrogel components, hydrogels that involve defined macroscale design can be challenging to generate on greater scales. One of the most difficult aspects of scaling hydrogel products is preserving sterility, which is mandatory for commercialization. Because of the higher amount of water in hydrogels, typical sterilization processes such as autoclaving are difficult or impossible to use without destroying the product. Sterilization of components and processes prior to hydration is the only feasible option. However, the steps involved in the sterilization process need to be carried out under an aseptic environment, which is a significant process hurdle. Filtration, radiation (gamma rays and e-beams), and heating operations are all examples of sterilizing techniques; at least one of these procedures must be compatible/non-destructive for the numerous components of a novel hydrogel therapy. There are plenty of other translation hazards and challenges that can be identified initially in the design process. Additionally, hydrogel formation that occurs via effortless mixing technique and self-assembly possibly will have a benefit in the course of scaled-up production [198]. The majority of biomedical materials research focuses on clinical translation, and the area has gathered publications that meticulously detail the therapeutic efficacy and processes of innovative biomaterials such as hydrogels. Enhanced transparency present in biomaterial production capabilities could highlight current constraints, uplifting their significance and enabling research and funding to address them.

## 7. Potential Applications of Hydrogel in the Biomedical Sector

Hydrogels are versatile products with wide and multifunctional applications in the biomedical sector. The upsurge of hydrogel technologies has created substantial contributions in biomedical applications that influence day-to-day life. Hydrogels, for example, have created a new class of optically adjustable soft materials in the form of soft contact lenses, making one of the most apparent helps to modern life. Hydrogel dressings are excellent for wound repair as they expedite the healing process by delivering moist and hospitable conditions for wounds through which easy breathing of the wound is possible with proper draining of exudate. Furthermore, these dressings are more comfortable and have a cooling effect. Hydrogels for tissue engineering purposes must have sufficient pore size to accept living cells, or they may be designed to dissolve or disintegrate over time, releasing growth factors and aiding in pore creation for living cells to enter and proliferate [199].

### 7.1. Contact Lenses

**Figure 5:**
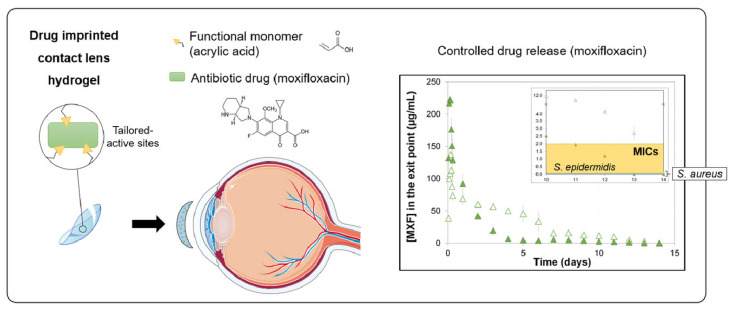
Drug-loaded contact lens hydrogel [201].

Contact lenses, a transparent or colored thin film, are widely used worldwide for better appearance, correcting refractive errors, less restriction, aesthetic reasons, and for the treatment of various diseases. These lenses can be hard or soft based on elastic behavior [200]. Many researchers have prepared hydrogels as contact lenses using natural as well as synthetic polymers loaded with antibiotics (Figure 5) [201]. Formulations of hydrogels have been designed using 2-methacryloyloxyethyl phosphorylcholine polymer as a crosslinking bioinspired agent with silicone to modify the surface of silicone and prepare a soft gel layer. The prepared formulation was found to have excellent lubricity, hydrophilicity, and flexibility due to a higher captive bubble contact angle. In addition to this, the coefficient of friction was reduced and the interaction force of protein deposition was lowered. All these properties of hydrogel contact lenses make it an excellent ocular formulation for the treatment of various diseases [202]. A combination of two different classes of drugs, such as an antibiotic (moxifloxacin hydrochloride) and anti-inflammatory (diclofenac sodium), were utilized with silicone and other natural polymers such as hyaluronate, alginate, and poly-lysine to prepare contact lens hydrogel. Molecular imprinting led to modifications in the release property of the formulation with non-toxicity, no ocular irritancy, and reduced cellular adhesion [203]. Various new observations in the reported literature reveal that hydrogel contact lenses have benefits because of their desirable features such as oxygen permeability, tunability, or flexibility. These contact lenses have the potential to be developed as an effective platform to overcome the limitation associated with conventional therapy. Varied impediments still remain to be solved pertaining to more efficacy, safety, and comfort in order to achieve successful and convenient ophthalmic hydrogels.

### 7.2. Wound Dressings

**Figure 6:**
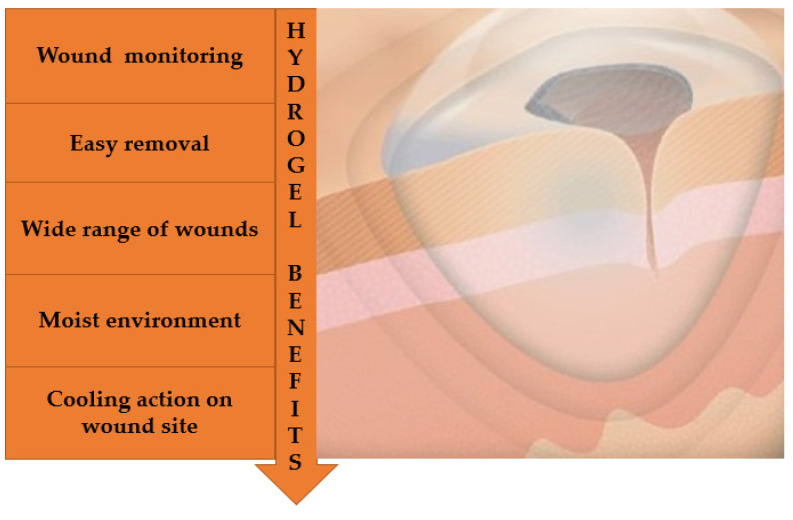
Benefits of hydrogel in wound healing.

A wound is a break or a defect that can occur due to any trauma or other physiological condition. It can be classified into acute or chronic wound categories based on healing duration [204,205]. Hydrogels are one of the moist dressings (Figure 6) and debriding agents for wound care. The moisture donor effect of hydrogels increases collagenase production and helps autolytic debridement to the granulating cavity and necrotic wounds [206]. Antibacterial drug-loaded aramid nanofiber (ANFs) hydrogels as a wound dressing have good water content, and high water retention ability (after incubating at RH 30% for 8 h), along with outstanding mechanical properties and high water adsorption properties. Besides these characteristics, antibacterial and anti-infective ANFs hydrogels were found to be non-cytotoxic and possess good hemolytic potential [207].

Chitosan (CA) conjugated with L-arginine, benzaldehyde group functionalized poly (ethylene glycol) (CHO-PEG-CHO), and polydopamine nanoparticles (pDA-NPs) were utilized for the formation of CA-pDA hydrogels. The hydrogels displayed good biocompatibility and adhesion with porous architecture and self-healing properties. Results revealed that integration of pDA-NPs help in prompt wound repair. Furthermore, CA-pDA hydrogels expedited healing action with minimized scar formation. The designed system could have immense potential in the clinic as wound dressings [208]. Multifunctional poly (citrate-glycol-siloxane)-based (PCGS) molecular hybrid hydrogel was developed by Cheng et al. The polymeric-based hydrogel showed better antibacterial, mechanical, and cytocompatibility properties against severe wounds. In vivo study confirmed effective wound healing activity for treating multidrug-resistant bacterial infection [209]. Gelatin methacrylate (GelMA) hybrid hydrogels were synthesized using lipopeptide surfactant (SF). The obtained hydrogels were effective for type I diabetic wounds and promoted angiogenesis via regulating macrophage polarization [210]. Additionally, the designed hydrogels by Shanmugapriya et al. conjugated with epidermal growth factor receptor showed good biocompatibility and cell proliferative property towards the targeted cells, but also displayed great ability to improve partial thickness in gastric ulcer healing and prevent scar formation [211]. A hydrogel using aldehyde functionalized sodium alginate with the help of Schiff base reaction and carboxymethyl chitosan (CS) was designed by Xuan et al. Short nanofibers of carboxymethyl-functionalized polymethyl methacrylate (PMAA) were made from sodium hydroxide-treated polymethyl methacrylate nanofibers and added to a CS solution. The nanofiber hydrogels depicted excellent self-healing action and significantly facilitated wound healing [212]. Injectable hydrogels are the other important hydrogel category that are very advantageous for skin tissue engineering, but due to their incompatibility of required hair follicle formation or induced infection, the antibacterial hydrogel was designed using Ag29 nanoclusters, mangiferin, and chitosan molecules for wound healing. The developed hydrogel depicted adequate swelling, decent injectability, superior biocompatibility, good degradability, and the regeneration of capillary vessels for wound healing [213]. Therefore, hydrogels have been found to have a positive impact on wound dressings because of excellent features such as great biocompatibility, high moisture retention, and activation of immune cells to accelerate wound healing. Recent advancements in hydrogel synthesis have enabled scientists to produce suitable wound dressing materials and ultimately better tissue regeneration. Despite substantial progress, more attention should be paid to the manufacturing aspects of hydrogels using innovative chemical and physical crosslinking, or a combination of the two, to better mimic in vivo dynamic behavior.

### 7.3. Tissue Engineering

**Figure 7:**
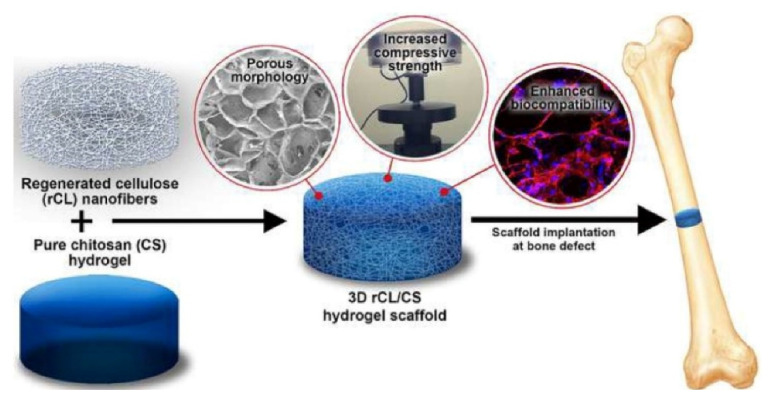
Hydrogel in bone tissue engineering [219].

Hydrogels are extensively used as agents for filling space in the tissue engineering area, as drug or bioactive substance delivery vehicles [214,215]. The formulated hydrogel of hyaluronic acid-tyramine (HA-Tyr) with silk-fibroin loaded with anabolic and anti-inflammatory drugs exhibited more sustained release behavior, which is essential for osteoarthritic joint treatment [216]. Researchers have developed a gelatin-graft-polyaniline/periodate-oxidized alginate hydrogel using polyethyleneimine with superior properties. The prepared injectable electroconductive hydrogels can also be approached for neural tissue engineering due to their advantageous properties such as cell adhesion and cell proliferation [217]. A UV irradiation method was adopted to prepare the non-swellable polymeric hydrogels by Ding et al. Injectable pentenyl chitosan hydrogels produced via N-acylation reaction was found to be hydrophobic, non-cytotoxic, and with no side effects, which made them favorable as a smart biomaterial and for biological tissue engineering [218]. Hydrogel scaffolds of chitosan (CS) and regenerated cellulose (rCL) nanofibers were prepared with unique porous morphology. The regeneration of cellulose acetate derivative via deacetylation technique shows high compressive strength. The rCL/CS scaffolds represent enhanced proliferation with osteogenic differentiation ability, biomineralization, and pre-osteoblast cell (MC3T3-E1) viability. All these potential applications of hydrogel scaffolds make it a prominent formulation for bone tissue engineering (Figure 7) [219]. Similarly, 3D printed hybrid scaffolds have been designed, comprised of alginate, gelatin, and carbon nanofibers. The hybrid composite scaffolds were chemically and mechanically better. Additions of nanofibers were found profitable for cellular proliferation and osteogenic differentiation. Therefore, the designed formulation possesses the potential for bone tissue engineering [220].

A recombinant human collagen and carboxymethyl chitosan mixture produced the soft hydrogel scaffolds by crosslinking-induced gelation technique. The fabricated hydrogels displayed strong biocompatibility with no cytotoxicity [221]. Other natural polysaccharides impregnated hydrogels such as silk fibroin (SF) and graphene oxide (GO) added hydrogels were also found suitable for bone tissue engineering. The hydrogels were prepared by enzyme-catalyzed crosslinking of SF and GO and then characterized fully. Moreover, bone marrow stromal cells (BMSCs) were encapsulated, which also proved the capability of BMSCs to differentiate and proliferate. Therefore, such an injectable BMSC hydrogel is considered a choice in the field of bone tissue engineering [222]. The composition of poly (γ-glutamic acid) (γ-PGA) and Fe^3^^+^ ligand double network hydrogel promoted BMSC proliferation and repair cartilage defect [223]. Fabrication of a gradient hydrogel scaffold via the moving photomask, with the help of poly (γ-glutamic acid) and chondroitin sulfate, was conducted by Liu et al [224]. The hydrogel displayed huge toughness and strength properties in addition to showing higher cell compatibility. Findings of in vitro stem cell differentiation depicted that light duration directly impacted the differentiation extent of stem cells, indicating that the designed hydrogel scaffold has the potential of simulating the function of natural cartilage, and therefore it can be employed for cartilage tissue engineering [224]. Several newer findings confirmed that designed hydrogels, owing to their inherent and fascinating characteristics, could be employed significantly for tissue engineering. Hydrogel scaffolds appear to signify an attractive strategy with unique observations in cartilage tissue engineering, bone tissue engineering, and similar. Hence, it has been concluded that hydrogels employed in the tissue engineering area must meet a number of design criteria to replicate the extracellular matrix and subsequently to function appropriately and promote the formation of new tissue. These hydrogels should present an innovative architecture for cellular proliferation. The design strategy must include both mechanical and physicochemical parameters along with consideration of biological performance. In addition, when producing hydrogel scaffolds for tissue engineering, factors such as accessibility and commercial viability should also be considered. Therefore, advancement in hydrogel platforms for versatile tissue engineering applications appears to represent a significant technology with ample fascinating opportunities. Consequently, it can be clearly concluded from all the scientific studies that hydrogels or their scaffolds are promising materials for tissue engineering applications.

## 8. Conclusions and Future Prospects

Over the last few decades, hydrogels have captured a lot of attention of researchers worldwide and are being extensively investigated as unique drug delivery systems. Significant advancements of hydrogels in site-specific delivery have been enormous due to their ease of modification with various polymeric materials and targeting ligands that result in tailored properties optimal for drug delivery. Hydrogels provide an encouraging platform for the delivery of useful therapeutic agents in various disorders including eye infection, cancer, rheumatoid arthritis, and fungal infections. Furthermore, novel designed hydrogels also display enhanced mechanical strength, therefore improving on flaws of traditional hydrogels and increasing the potential role of smart hydrogels in some concerns, such as the controlled swelling rate of hydrogels, while increasing their mechanical characteristics, matching the dimension requirements of tissues besides organs. Biocompatibility enhancement is also required for achieving simulation of the extracellular matrix structure and functions in hydrogels. Hydrogel degradation rate should be adjustable to match tissue-specific mechanical characteristics in addition to regeneration requirements, and to produce the complex structural and functional components required to behave as organ substitutes, hydrogels need to be coupled with different efficacious materials. Despite the fact that numerous formulations of hydrogels are used clinically, there is still scope and opportunities for further perfection and effectiveness. Progress in biomaterials has also broadened the hydrogel range intended for drug delivery in a controlled manner. The hydrogels may become outstanding drug delivery vehicles with slight modifications to the existing ones, surpassing the drawbacks and current constraints of several traditional delivery forms and delivering drugs efficiently for various diseases. Ongoing research will eventually address drawbacks, leading to a pioneering new paradigm in drug delivery, bioengineering, and advances in tissue replacement, in addition to regeneration.

## Figures and Tables

**Table 1:** Hydrogel loaded with various active ingredients.

Therapeutically Active Substances/Drugs	Polymers/ Monomers	Method/Mechanism	Inference	References
Adipose stem cells	Hyaluronic acid	Ultraviolet irradiation	Promising carrier for stem cell delivery in wound repair and skin tissue engineering	[124]
Vancomycin	Agarose	Hydrogen bonding	Potential candidate for healing of infected wounds	[125]
Catechol	Chitosan	Covalent bonding	Designed system utilized successfully for buccal drug delivery	[126]
Diacerein	Polyethylene glycol	Hydrophobic interactions	Hydrogel successfully repaired spinal cord injury	[127]
Heparin	Poloxamer 407	Cold method	Delivery system provided release in controlled manner and can be employed for nerve regeneration	[128]

**Table 2:** Recent patents on hydrogels.

Patent No	Title	Highlights	Date and Reference
US20210023121A1	Thrombin-responsive hydrogels and devices for auto-anticoagulant regulation	Compounds, compositions, devices, and methods for auto-anticoagulation regulation. Further disclosed methods for treating or preventing thrombosis	28 January2021 [161]
US20200206030A1	High-precision drug delivery by dual-domain ocular device	Nanocomposite ocular device provided controlled and sustained release of the drug	2 July 2020 [162]
US20200214882A1	Treating conditions caused by abnormal growth of pathogens in body cavities	The invention based on cooling or temperature reducing treatment device to cure vaginal infections, including vulvovaginal candidiasis (VVC), bacterial vaginosis (BV), and to reduce biofilms	9 July 2020 [163]
US20200214886A1	Programmable therapeutic agent delivery from eye mounted device	Systems and methods for on-demand delivery of a therapeutic agent from an eye mounted device	9 July 2020 [164]
US20200214887A1	Eye mounted device for therapeutic agent release	Devices and systems for targeted and controlled delivery of a therapeutic agent to a treatment site of an eye	9 July 2020 [165]
US20200215136A1	Medication dispensing system	Method disclosed for providing treatment with cannabis to treat insomnia, anxiety, or seizure, among others	9 July 2020 [166]
US20200215194A1	Apoptosis inhibitor formulations for prevention of hearing loss	Formulation for sustained release of an apoptosis inhibitor in the inner ear to protect from hearing loss, especially due to exposure to chemotherapy with drugs such as cisplatin	9 July 2020 [167]
US20200282062A1	Medication	Method of treating seizure, insomnia, or anxiety includes administering, via a patch, pill, lotion, mist, or hydrogel to humans	10 September 2020 [168]
US20200316052A1	Ophthalmic composition	Methods of arresting or preventing myopia development by administering to an eye of an individual an effective amount of an ophthalmic composition	27 September 2020 [169]
US20200330380A1	Biocompatible organogel matrices for intraoperative preparation of a drug delivery depot	Disclosure directed to an organogel drug depot for use in delivering an active agent to a surgical site, such as an orthopedic implant site	22 October 2020 [170]

**Table 3:** Clinical trials on hydrogels.

Condition	Hydrogel	Clinical Trial Number	Status	Reference
Osteoarthritis, knee pain	Hydrogel injection	NCT04061733	Recruiting	[176]
Ruptured aneurysm	Second-generation hydrogel coils	NCT03252314	Recruiting	[177]
Wound infection, surgical	Pico^®^ negative pressure dressing, Aquacel Surgical^®^ hydrogel dressing	NCT04265612	Recruiting	[178]
Refractive error-myopia bilateral	Hioxifilcon A standard hydrogel contact lens with hyaluronic acid (HA)	NCT04671108	Recruiting	[179]
Wound healing	3% sodium pentaborate pentahydrate	NCT02241811	Recruiting	[180]
Mucositis oral, head and neck cancer	MucoLox, sodium bicarbonate	NCT03461354	Recruiting	[181]
hypervascular tumors	Instylla HES, TAE or cTACE	NCT04523350	Recruiting	[182]
osteoarthritis, knee	PAAG-OA, Synvisc-One	NCT04045431	Recruiting	[183]
myopia	OxyAqua, Si-Hy	NCT03139201	Completed	[184]
Cancer of the prostate	PEG hydrogel (SpaceOAR)	NCT02212548	Completed	[185]
Cerebral aneurysm	Hydro coil embolic system, control (bare platinum coils)	NCT01407952	Completed	[186]
Astigmatism	verofilcon atoric contact lenses	NCT04464044	Completed	[187]
Knee osteoarthritis	PVA hydrogel, Synvisk-One^®^	NCT04693104	Completed	[188]
Bladder carcinoma	Polyethylene glycol hydrogel	NCT03125226	Completed	[189]
Myopia	Somofilcon A, Omafilcon A-Proclear (PC)	NCT03098745	Completed	[190]
Ametropia	Methafilcon A toric contact lenses, fanfilcon A toric contact lenses	NCT03835221	Completed	[191]
Intracranial aneurysm subarachnoid hemorrhage	Hydrogel coil, Bare platinum coil	NCT01516658	Completed	[192]

## Data Availability

Not applicable.

## References

[1] Emilian L.S. (2010). Nanotechnology for delivery of drugs and biomedical applications. Curr. Clin. Pharmacol..

[2] Wen H., Jung H., Li X. (2015). Drug delivery approaches in addressing clinical pharmacology-related issues: Opportunities and challenges. AAPS J..

[3] Peppas N.A., Hilt J.Z., Khademhosseini A., Langer R. (2006). Hydrogels in biology and medicine: From molecular principles to bionanotechnology. Adv. Mater..

[4] Schoenmakers D.C., Rowan A.E., Kouwer P.H.J. (2018). Crosslinking of fibrous hydrogels. Nature Commun..

[5] Varaprasad K., Raghavendra G.M., Jayaramudu T., Yallapu M.M., Sadiku R. (2017). A mini review on hydrogels classification and recent developments in miscellaneous applications. Mater. Sci. Eng. C.

[6] Khalesi H., Lu W., Nishinari K., Fang Y. (2020). New insights into food hydrogels with reinforced mechanical properties: A review on innovative strategies. Adv. Colloid Interface Sci..

[7] Dreiss C.A. (2020). Hydrogel design strategies for drug delivery. Curr. Opin. Colloid Interface Sci..

[8] Abdollahiyan P., Baradarn B., de la Guardia M., Oroojalian F., Mokhtarzadeh A. (2020). Cutting-edge progress and challenges in stimuli responsive hydrogel microenvironment for success in tissue engineering today. J. Control. Release.

[9] Zhang Y.S., Khademhosseini A. (2017). Advances in engineering hydrogels. Science.

[10] Pawar A.A., Saada G., Cooperstein I., Larush L., Jackman J.A., Tabaei S.R., Cho N.J., Magdassi S. (2016). High-performance 3D printing of hydrogels by water-dispersible photo initiator nanoparticles. Sci. Adv..

[11] Bashir S., Hina M., Iqbal J., Rajpar A.H., Mujtaba M.A., Alghamdi N.A., Wageh S., Ramesh K., Ramesh S. (2020). Fundamental concepts of hydrogels: Synthesis, properties, and their applications. Polymers.

[12] Catoira M.C., Fusaro L., Di Francesco D., Ramella M., Boccafoschi F. (2019). Overview of natural hydrogels for regenerative medicine applications. J. Mater. Sci. Mater. Med..

[13] Ahmed E.M. (2015). Hydrogel: Preparation, characterization, and applications: A review. J. Adv. Res..

[14] Khan S., Ullah A., Ullah K., Rehman N. (2016). Insight into hydrogels. Des. Monomers Polym..

[15] Baroli B. (2007). Hydrogels for tissue engineering and delivery of tissue-inducing substances. J. Pharm. Sci..

[16] Entezami A.A., Massoumi B. (2006). Artificial muscles, biosensors and drug delivery systems based on conducting polymers: A review. Iran. Polym. J..

[17] El-Sherbiny I.M., Yacoub M.H. (2013). Hydrogel scaffolds for tissue engineering: Progress and challenges. Glob. Cardiol. Sci. Pract..

[18] Wheeler J.C., Woods J.A., Cox M.J., Cantrell R.W., Watkins F.H., Edlich R.F. (1996). Evolution of hydrogel polymers as contact lenses, surface coatings, dressings, and drug delivery systems. J. Long-Term Eff. Med. Implant..

[19] Amin S., Rajabnezhad S., Kohli K. (2009). Hydrogels as potential drug delivery systems. Sci. Res. Essays..

[20] Aroca A.S., Ribelles J.L.G., Pradas M.M., Garayo A.V., Antón J.S. (2007). Characterisation of macroporous poly (methyl methacrylate) coated with plasma-polymerised poly (2-hydroxyethyl acrylate). Eur. Polym. J..

[21] Rowley J.A., Madlambayan G., Mooney D.J. (1999). Alginate hydrogels as synthetic extracellular matrix materials. Biomaterials.

[22] Liu Q., Hedberg E.L., Liu Z., Bahulekar R., Meszlenyi R.K., Mikos A.G. (2000). Preparation of macroporous poly (2-hydroxyethyl methacrylate) hydrogels by enhanced phase separation. Biomaterials.

[23] Oh K.S., Han S.K., Choi Y.W., Lee J.H., Lee J.Y., Yuk S.H. (2004). Hydrogen-bonded polymer gel and its application as a temperature-sensitive drug delivery system. Biomaterials.

[24] Baroli B.M. (2006). Photopolymerization in drug delivery, tissue engineering and cell encapsulation: Issues and potentialities. J. Chem. Technol. Biotechnol..

[25] Kimura M., Fukumoto K., Watanabe J., Ishihara K. (2004). Hydrogen-bonding-driven spontaneous gelation of water-soluble phospholipid polymers in aqueous medium. J. Biomater. Sci. Polym. Ed..

[26] Stenekes R.J.H., Talsma H., Hennink W.E. (2001). Formation of dextran hydrogels by crystallization. Biomaterials.

[27] Aalaie J., Vasheghani-Farahani E., Rahmatpour A., Semsarzadeh M.A. (2008). Effect of montmorillonite on gelation and swelling behavior of sulfonated polyacrylamide nanocomposite hydrogels in electrolyte solutions. Eur. Polym. J..

[28] Qiu Y., Park K. (2001). Environment-sensitive hydrogels for drug delivery. Adv. Drug Deliv. Rev..

[29] Cappello J., Crissman J.W., Crissman M., Ferrari F.A., Textor G., Wallis O., Whitledge J.R., Zhou X., Burman D., Aukerman L. (1998). In-situ self-assembling protein polymer gel systems for administration, delivery, and release of drugs. J. Control. Release.

[30] Ganji F., Abdekhodaie M.J., Ahmed Ramazani S.A. (2007). Gelation time and degradation rate of chitosan-based injectable hydrogel. J. Solgel. Sci. Technol..

[31] Mohamadnia Z., Jamshidi A., Mobedi H., Zohourian M.M., Ahmadi E. (2007). Full natural hydrogel beads for controlled release of acetate and disodium phosphate derivatives of betamethasone. Iran. Polym. J..

[32] Satish C.S., Satish K.P., Shivakumar H.G. (2006). Hydrogels as controlled drug delivery systems: Synthesis, crosslinking, water and drug transport mechanism. Indian J. Pharm. Sci..

[33] Hennink W.E., van Nostrum C.F. (2012). Novel crosslinking methods to design hydrogels. Adv. Drug Deliv. Rev..

[34] Lopes C.M.A., Felisberti M.I. (2003). Mechanical behaviour and biocompatibility of poly (1-vinyl-2-pyrrolidinone)–gelatin IPN hydrogels. Biomaterials.

[35] Oh E.J., Kang S.W., Kim B.S., Jiang G., Cho I.H., Hahn S.K. (2008). Control of the molecular degradation of hyaluronic acid hydrogels for tissue augmentation. J. Biomed. Mater. Res. A.

[36] Coviello T., Grassi M., Rambone G., Alhaique F. (2001). A crosslinked system from scleroglucan derivative: Preparation and characterization. Biomaterials.

[37] Wang Z., Zhou X., Mao Z., Ye R., Mo Y., Finlow D.E. (2008). Synthesis and characterization of biodegradable poly (lactic acid-co-glycine) via direct melt copolymerization. Iran. Polym. J..

[38] Bagheri S., Mohammadi R.D.E.H.J., Hassan A. (2007). Synthesis and characterization of biodegradable random copolymers of L-lactide, glycolide and trimethylene carbonate. Iran. Polym. J..

[39] Garcia Y., Collighan R., Griffin M., Pandit A. (2007). Assessment of cell viability in a three-dimensional enzymatically cross-linked collagen scaffold. J. Mater. Sci. Mater. Med..

[40] Zhang Y., Zhu W., Ding J. (2005). Preparation of thermosensitive microgels via suspension polymerization using different temperature protocols. J. Biomed. Mater. Res. A.

[41] McKenzie M., Betts D., Suh A., Bui K., Kim L.D., Cho H. (2015). Hydrogel-based drug delivery systems for poorly water-soluble drugs. Molecules.

[42] Sakai S., Ueda K., Taya M. (2015). Peritoneal adhesion prevention by a biodegradable hyaluronic acid-based hydrogel formed in situ through a cascade enzyme reaction initiated by contact with body fluid on tissue surfaces. Acta Biomater..

[43] Xinming L., Yingde C., Lloyd A.W., Mikhalovsky S.V., Sandeman S.R., Howel C.A., Liewen L. (2008). Polymeric hydrogels for novel contact lens-based ophthalmic drug delivery systems: A review. Cont. Lens Anterior Eye.

[44] Sim S., Figueiras A., Veiga F. (2012). Modular hydrogels for drug delivery. J. Biomater. Nanobiotechnol..

[45] Hoare T.R., Kohane D.S. (2008). Hydrogels in drug delivery: Progress and challenges. Polymer.

[46] Chai Q., Jiao Y., Yu X. (2017). Hydrogels for biomedical applications: Their characteristics and the mechanisms behind them. Gels.

[47] Larrañeta E., Stewart S., Ervine M., Al-Kasasbeh R., Donnelly R.F. (2018). Hydrogels for hydrophobic drug delivery, classification, synthesis and applications. J. Funct. Biomater..

[48] Li J., Mooney D.J. (2016). Designing hydrogels for controlled drug delivery. Nat. Rev. Mater..

[49] Chirani N., Gritsch L., Motta F.L., Fare S. (2015). History and applications of hydrogels. J. Biomed. Sci..

[50] Ullah F., Othman M.B.H., Javed F., Ahmad Z., Akil H.M. (2015). Classification, processing and application of hydrogels: A review. Mater. Sci. Eng. C.

[51] Hu H., Xu F.J. (2020). Rational design and latest advances of polysaccharide-based hydrogels for wound healing. Biomater. Sci..

[52] Zhao M., Tang Z., Zhang X., Li Z., Xiao H., Zhang M., Liu K., Ni Y., Huang L., Chen L. (2020). A self-healing, stretchable, and conductive poly (N-vinylpyrrolidone)/gallic acid composite hydrogel formed via hydrogen bonding for wearable electronic sensors. Compos. Sci. Technol..

[53] Elsayed M.M. (2019). Hydrogel preparation technologies: Relevance kinetics, thermodynamics and scaling up aspects. J. Polym. Environ..

[54] Yuan N., Xu L., Xu B., Zhao J., Rong J. (2018). Chitosan derivative-based self-healable hydrogels with enhanced mechanical properties by high-density dynamic ionic interactions. Carbohydr. Polym..

[55] Wu J., Gong X., Fan Y., Xia H. (2011). Physically crosslinked poly (vinyl alcohol) hydrogels with magnetic field controlled modulus. Soft Matter.

[56] Ding L., Song S., Chen L., Shi J., Zhao B., Teng G., Zhang J. (2021). A freeze-thawing method applied to the fabrication of 3-d curdlan/polyvinyl alcohol hydrogels as scaffolds for cell culture. Int. J. Biol. Macromol..

[57] Lim J.Y.C., Lin Q., Xue K., Loh X.J. (2019). Recent advances in supramolecular hydrogels for biomedical applications. Mater. Tod. Adv..

[58] Takei T., Yoshihara R., Danjo S., Fukuhara Y., Evans C., Tomimatsu R., Ohzuno Y., Yoshida M. (2020). Hydrophobically-modified gelatin hydrogel as a carrier for charged hydrophilic drugs and hydrophobic drugs. Int. J. Biol. Macromol..

[59] Jiang L., Luo Z., Loh X.J., Wu Y.L., Li Z. (2019). PHA-based thermogel as a controlled zero-order chemotherapeutic delivery system for the effective treatment of melanoma. ACS App. BioMater..

[60] Ma X., Zhao Y. (2015). Biomedical applications of supramolecular systems based on host-guest interactions. Chem. Rev..

[61] Yang H., Yuan B., Zhang X., Scherman O.A. (2014). Supramolecular chemistry at interfaces: Host-guest interactions for fabricating multifunctional biointerfaces. Acc. Chem. Res..

[62] Frisch H., Besenius P. (2015). pH-switchable self-assembled materials. Macromol. Rapid Commun..

[63] Qi Z., Schalley C.A. (2014). Exploring macrocycles in functional supramolecular gels: From stimuli responsiveness to systems chemistry. Acc. Chem. Res..

[64] Mantooth S.M., Munoz-Robles B.G., Webber M.J. (2019). Dynamic hydrogels from host–guest supramolecular interactions. Macro. Bios..

[65] Akhtar M.F., Hanif M., Ranjha N.M. (2016). Methods of synthesis of hydrogels: A review. Saudi. Pharm. J..

[66] Yu F., Cao X., Du J., Wang G., Chen X. (2015). Multifunctional hydrogel with good structure integrity, self-healing, and tissue-adhesive property formed by combining diels-alder click reaction and acylhydrazone bond. ACS Appl. Mater. Interfaces.

[67] Ding F., Wu S., Wang S., Xiong Y., Li Y., Li B., Deng H., Du Y., Xiao L., Shi X. (2015). A dynamic and self-crosslinked polysaccharide hydrogel with autonomous self-healing ability. Soft Matter.

[68] Ma X., Xu T., Chen W., Qin H., Chi B., Ye Z. (2018). Injectable hydrogels based on the hyaluronic acid and poly (γ-glutamic acid) for controlled protein delivery. Carbohydr. Polym..

[69] Kalia J., Raines R.T. (2008). Hydrolytic stability of hydrazones and oximes. Angew. Chem. Int. Ed..

[70] Mukherjee S., Hill M.R., Sumerlin B.S. (2015). Self-healing hydrogels containing reversible oxime crosslinks. Soft Matter.

[71] Sui X., van Ingen L., Hempenius M.A., Vancso G.J. (2010). Preparation of a rapidly forming poly (ferrocenylsilane)-poly (ethylene glycol)-based hydrogel by a thiol-michael addition click reaction. Macromol. Rapid Commun..

[72] Rizzi S.C., Hubbell J.A. (2005). Recombinant protein-co-PEG networks as cell-adhesive and proteolytically degradable hydrogel matrixes. Part I: Development and physicochemical characteristics. Biomacromolecules.

[73] Bhattarai Gunn J., Zhang M. (2010). Chitosan-based hydrogels for controlled, localized drug delivery. Adv. Drug Deliv. Rev..

[74] Guo J., Zhang Y., Yang X.Q. (2012). A novel enzyme cross-linked gelation method for preparing food globular protein-based transparent hydrogel. Food Hydrocoll..

[75] Chen F., Yu S., Liu B., Ni Y., Yu C., Su Y., Zhu X., Yu X., Zhou Y., Yan D. (2016). An injectable enzymatically crosslinked carboxymethylated pullulan/chondroitin sulfate hydrogel for cartilage tissue engineering. Sci. Rep..

[76] Hasturk O., Jordan K.E., Choi J., Kaplan D.L. (2020). Enzymatically crosslinked silk and silk-gelatin hydrogels with tunable gelation kinetics, mechanical properties and bioactivity for cell culture and encapsulation. Biomaterials.

[77] Mozalewska W., Czechowska-Biskup R., Olejnik A.K., Wach R.A., Ulański P., Rosiak J.M. (2017). Chitosan-containing hydrogel wound dressings prepared by radiation technique. Radiat. Phys. Chem..

[78] Relleve L.S., Gallardo A.K.R., Tecson M.G., Luna J.A. (2021). Biocompatible hydrogels of carboxymethyl hyaluronic acid prepared by radiation-induced crosslinking. Radiat. Phys. Chem..

[79] Perera M.M., Ayres N. (2020). Dynamic covalent bonds in self-healing, shape memory, and controllable stiffness hydrogels. Polym. Chem..

[80] Liechty W.B., Kryscio D.R., Slaughter B.V., Peppas N.A. (2010). Polymers for drug delivery systems. Annu. Rev. Chem. Biomol. Eng..

[81] Tabata Y. (2009). Biomaterial technology for tissue engineering applications. J. R. Soc. Interface.

[82] Shantha K.L., Harding D.R.K. (2002). Synthesis and evaluation of sucrose-containing polymeric hydrogels for oral drug delivery. J. Appl. Polym. Sci..

[83] Koczkur K.M., Mourdikoudis S., Polavarapu L. (2015). Polyvinylpyrrolidone (PVP) in nanoparticle synthesis. Dalton Trans..

[84] Kurakula M., Rao G.S.N.K. (2020). Moving polyvinyl pyrrolidone electrospun nanofibers and bioprinted scaffolds toward multidisciplinary biomedical applications. Eur. Polym. J..

[85] Haaf F., Sanner A., Straub F. (1985). Polymers of N-vinylpyrrolidone: Synthesis, characterization and uses. Polym. J..

[86] Ajji Z., Maarouf M., Khattab A., Ghazal H. (2020). Synthesis of pH-responsive hydrogel based on PVP grafted with crotonic acid for controlled drug delivery. Radiat. Phys. Chem..

[87] Demeter M., Meltzer V., Călina I., Scărișoreanu A., Micutz M., Kaya M.G. (2020). Highly elastic superabsorbent collagen/PVP/PAA/PEO hydrogels crosslinked via e-beam radiation. Radiat. Phys. Chem..

[88] Amer L.D., Saleh L.S., Walker C., Thomas S., Janssen W.J., Alper S., Bryant S.J. (2019). Inflammation via myeloid differentiation primary response gene 88 signaling mediates the fibrotic response to implantable synthetic poly (ethylene glycol) hydrogels. Acta Biomater..

[89] Petit A., Redout E.M., Van de Lest C.H., de Grauw J.C. (2015). Sustained intra-articular release of celecoxib from in situ forming gels made of acetyl-capped PCLA-PEG-PCLA triblock copolymers in horses. Biomaterials.

[90] Akalin O.B., Bayraktar H. (2020). Alteration of cell motility dynamics through collagen fiber density in photopolymerized polyethylene glycol hydrogels. Int. J. Biol. Macromol..

[91] Jafari A., Hassanajili S., Azarpira N., Karimi M.B., Geramizadeh B. (2019). Development of thermal-crosslinkable chitosan/maleic terminated polyethylene glycol hydrogels for full thickness wound healing: In vitro and in vivo evaluation. Eur. Polym. J..

[92] Raina N., Pahwa R., Khosla J.K., Gupta P.N., Gupta M. (2021). Polycaprolactone-based materials in wound healing applications. Polym. Bull..

[93] Sabzi M., Afshari M.J., Babaahmadi M., Shafagh N. (2020). pH-dependent swelling and antibiotic release from citric acid crosslinked poly (vinyl alcohol) (PVA)/nano silver hydrogels. Colloids Surf. B.

[94] Nurkeeva Z.S., Khutoryanskiy V.V., Mun G.A., Sherbakova M.V., Ivaschenko A.T., Aitkhozhina N.A. (2004). Polycomplexes of poly (acrylic acid) with streptomycin sulfate and their antibacterial activity. Eur. J. Pharm. Biopharm..

[95] Mallawarachchi S., Mahadevan A., Gejji V., Fernando S. (2019). Mechanics of controlled release of insulin entrapped in polyacrylic acid gels via variable electrical stimuli. Drug Deliv. Transl. Res..

[96] Nath J., Chowdhury A., Ali I., Dolui S.K. (2019). Development of a gelatin-g-poly (acrylic acid-co-acrylamide)–montmorillonite superabsorbent hydrogels for in vitro controlled release of vitamin B12. J. Appl. Polym. Sci..

[97] Xu X., Liu Y., Fu W., Yao M., Ding Z., Xuan J., Li D., Wang S. (2020). Poly (N-isopropylacrylamide)-based thermoresponsive composite hydrogels for biomedical applications. Polymers.

[98] Zhang Z., Wang S., Waterhouse G.I.N., Zhang Q., Li L. (2020). Poly (N-isopropylacrylamide)/mesoporous silica thermosensitive composite hydrogels for drug loading and release. J. Appl. Polym. Sci..

[99] Khan S., Akhtar N., Minhas M.U., Badshah S.F. (2019). pH/thermo-dual responsive tunable in situ cross-linkable depot injectable hydrogels based on poly (N-isopropylacrylamide)/carboxymethyl chitosan with potential of controlled localized and systemic drug delivery. AAPS PharmSciTech.

[100] Kamaci M. (2020). Polyurethane-based hydrogels for controlled drug delivery applications. Eur. Polym. J..

[101] Wen J., Jia Z., Zhang X., Pan M., Yuan J., Zhu L. (2020). Tough., Thermo-responsive, biodegradable and fast self-healing polyurethane hydrogel based on microdomain-closed dynamic bonds design. Mater. Today Commun..

[102] Viezzer C., Mazzuca R., Machado D.C., Camargo Forte MM de Ribelles J.L. (2020). A new waterborne chitosan-based polyurethane hydrogel as a vehicle to transplant bone marrow mesenchymal cells improved wound healing of ulcers in a diabetic rat model. Carbohydr. Polym..

[103] Segura T., Anderson B.C., Chung P.H., Webber R.E., Shull K.R., Shea L.D. (2005). Crosslinked hyaluronic acid hydrogels: A strategy to functionalize and patte. Biomaterials.

[104] Patil R., Kansara V., Ray D., Aswal V.K., Jha P.K., Bahadur P., Tiwari S. (2019). Slow degrading hyaluronic acid hydrogel reinforced with cationized graphene nanosheets. Int. J. Biol. Macromol..

[105] Zhu M., Wang J., Li N. (2018). A novel thermo-sensitive hydrogel-based on poly (N-isopropylacrylamide)/hyaluronic acid of ketoconazole for ophthalmic delivery. Artif. Cells Nanomed. Biotechnol..

[106] Raina N., Rani R., Khan A., Nagpal K., Gupta M. (2020). Interpenetrating polymer network as a pioneer drug delivery system: A review. Polym. Bull..

[107] Jafari H., Atlasi Z., Mahdavinia G.R., Hadifar S., Sabzi M. (2021). Magnetic κ, carrageenan/chitosan/montmorillonite nanocomposite hydrogels with controlled sunitinib release. Mater. Sci. Eng. C.

[108] Chen Z., Yang M., Wang Q., Bai J., McAlinden C., Skiadaresi E., Zhang J., Pan L., Mei C., Zeng Z. (2021). Hydrogel eye drops as a non-invasive drug carrier for topical enhanced Adalimumab permeation and highly efficient uveitis treatment. Carbohydr. Polym..

[109] Raina N., Rani R., Pahwa R., Gupta M. (2020). Biopolymers and treatment strategies for wound healing: An insight view. Int. J. Polym. Mater. Polym. Biomater..

[110] Nazeri M.T., Javanbakht S., Shaabani A., Ghorbani M. (2020). 5-aminopyrazole-conjugated gelatin hydrogel: A controlled 5-fluorouracil delivery system for rectal administration. J. Drug Del. Sci. Tech..

[111] Akhlaq M., Azad A.K., Ullah I., Nawaz A., Safdar M., Bhattacharya T., Uddin A.B., Abbas S.A., Mathews A., Kundu S.K. (2021). Methotrexate-loaded gelatin and polyvinyl alcohol (Gel/PVA) hydrogel as a pH-sensitive matrix. Polymers.

[112] Thu H.E., Zulfakar M.H., Ng S.F. (2012). Alginate based bilayer hydrocolloid films as potential slow-release modern wound dressing. Int. J. Pharm..

[113] Afshar M., Dini G., Vaezifar S., Mehdikhani M., Movahedi B. (2020). Preparation and characterization of sodium alginate/polyvinyl alcohol hydrogel containing drug-loaded chitosan nanoparticles as a drug delivery system. J. Drug Del. Sci. Tech..

[114] Oliveira J.T., Santos T.C., Martins L., Picciochi R., Marques A.P., Castro A.G., Neves N.M., Mano J.F., Reis R.L. (2010). Gellan gum injectable hydrogels for cartilage tissue engineering applications: In vitro studies and preliminary in vivo evaluation. Tissue Eng. Part A.

[115] Gupta D., Tator C.H., Shoichet M.S. (2006). Fast-gelling injectable blend of hyaluronan and methylcellulose for intrathecal, localized delivery to the injured spinal cord. Biomaterials.

[116] Bukhari S.M.H., Khan S., Rehanullah M., Ranjha N.M. (2015). Synthesis and characterization of chemically cross-linked acrylic acid/gelatin hydrogels: Effect of pH and composition on swelling and drug release. Int. J. Polym. Sci..

[117] Amirian J., Linh N.T.B., Min Y.K., Lee B.T. (2015). The effect of BMP-2 and VEGF loading of gelatin-pectin-BCP scaffolds to enhance osteoblast proliferation. J. Appl. Polym. Sci..

[118] Jagur-Grodzinski J. (2010). Polymeric gels and hydrogels for biomedical and pharmaceutical applications. Polym. Adv. Technol..

[119] Bajpai M., Bajpai S.K., Gautam D. (2014). Investigation of regenerated cellulose/poly (acrylic acid) composite films for potential wound healing applications: A preliminary study. J. Appl. Chem..

[120] Chen D.T.N., Wen Q., Janmey P.A., Crocker J.C., Yodh A.G. (2010). Rheology of soft materials. Annu. Rev. Condens. Matter Phys..

[121] Yan C., Pochan D.J. (2010). Rheological properties of peptide-based hydrogels for biomedical and other applications. Chem. Soc. Rev..

[122] Olad A., Eslamzadeh M., Mirmohseni A. (2019). Physicochemical evaluation of nanocomposite hydrogels with covalently incorporated poly (vinyl alcohol) functionalized graphene oxide. J. Appl. Polym. Sci..

[123] Wang Y., Xue Y., Wang J., Zhu Y., Zhu Y., Zhang X., Liao J. (2019). A composite hydrogel with high mechanical strength, fluorescence, and degradable behavior for bone tissue engineering. Polymers.

[124] Ahovan Z.A., Khosravimelal S., Eftekhari B.S., Mehrabi S., Hashemi A., Eftekhari S., Milan M., Mobaraki P.B., Seifalian A.M., Gholipourmalekabadi M. (2020). Thermo-responsive chitosan hydrogel for healing of full-thickness wounds infected with XDR bacteria isolated from burn patients: In vitro and in vivo animal model. Int. J. Biol. Macromol..

[125] Deng H., Sun J., Yu Z., Guo Z., Xu C. (2021). Low-intensity near-infrared light-triggered spatiotemporal antibiotics release and hyperthermia by natural polysaccharide-based hybrid hydrogel for synergistic wound disinfection. Mater. Sci. Eng. C.

[126] Xu J., Strandman S., Zhu J.X.X., Barralet J., Cerruti M. (2015). Genipin-crosslinked catechol-chitosan mucoadhesive hydrogels for buccal drug delivery. Biomaterials.

[127] Zhang K., Li J., Jin J., Dong J., Li L., Xue B., Wang W., Jiang Q., Cao Y. (2020). Injectable, anti-inflammatory and conductive hydrogels based on graphene oxide and diacerein-terminated four-armed polyethylene glycol for spinal cord injury repair. Mater. Des..

[128] Li R., Li Y., Wu Y., Zhao Y., Chen H., Yuan Y., Xu K., Zhang H., Lu Y., Wang J. (2018). Heparin-poloxamer thermosensitive hydrogel loaded with bFGF and NGF enhances peripheral nerve regeneration in diabetic rats. Biomaterials.

[129] Liu L., Gao Q., Lu X., Zhou H. (2016). In situ forming hydrogels based on chitosan for drug delivery and tissue regeneration. Asian J. Pharm..

[130] Hamidi M., Azadi A., Rafiei P. (2008). Hydrogel nanoparticles in drug delivery. Adv. Drug Deliv. Rev..

[131] Azari A.A., Barney N.P. (2013). Conjunctivitis: A systematic review of diagnosis and treatment. JAMA.

[132] Deepthi S., Jose J. (2019). Novel hydrogel-based ocular drug delivery system for the treatment of conjunctivitis. Int. Ophthalmol..

[133] Ren N., Sun R., Xia K., Zhang Q., Li W., Wang F., Zhang X., Ge Z., Wang L., Fan C. (2019). DNA-based hybrid hydrogels sustain water-insoluble ophthalmic therapeutic delivery against allergic conjunctivitis. ACS Appl. Mater. Interfaces.

[134] Rendon A., Schäkel K. (2019). Psoriasis pathogenesis and treatment. Int. J. Mol. Sci..

[135] Kim A.R., Lee S.L., Park S.N. (2018). Properties and in vitro drug release of pH-and temperature-sensitive double cross-linked interpenetrating polymer network hydrogels based on hyaluronic acid/poly (N-isopropylacrylamide) for transdermal delivery of luteolin. Int. J. Biol. Macromol..

[136] Gabriel D., Mugnier T., Courthion H., Kranidioti K., Karagianni N., Denis M.C., Lapteva M., Kalia Y., Möller M., Gurny R. (2016). Improved topical delivery of tacrolimus: A novel composite hydrogel formulation for the treatment of psoriasis. J. Control. Release.

[137] Guo Q., Wang Y., Xu D., Nossent J., Pavlos N.J., Xu J. (2018). Rheumatoid arthritis: Pathological mechanisms and modern pharmacologic therapies. Bone Res..

[138] Oliveira I.M., Gonçalves C., Shin M.E., Lee S., Reis R.L. (2020). Anti-inflammatory properties of injectable betamethasone-loaded tyramine-modified gellan gum/silk fibroin hydrogels. Biomolecules..

[139] Yin N., Guo X., Sun R., Liu H., Tang L., Gou J., Yin T., He H., Zhang Y., Tang X. (2020). Intra-articular injection of indomethacin–methotrexate in situ hydrogel for the synergistic treatment of rheumatoid arthritis. J. Mater. Chem. B.

[140] Sharma G.N., Dave R., Sanadya J., Sharma P., Sharma K.K. (2010). Various types and management of breast cancer: An overview. J. Adv. Pharm. Technol. Res..

[141] Taleblou N., Sirousazar M., Hassan Z.M., Khaligh S.G. (2020). Capecitabine-loaded anti-cancer nanocomposite hydrogel drug delivery systems: In vitro and in vivo efficacy against the 4T1 murine breast cancer cells. J. Biomater. Sci. Polym. Ed..

[142] Qi Y., Min H., Mujeeb A., Zhang Y., Han X., Zhao X., Anderson G.J., Zhao Y., Nie G. (2018). Injectable hexapeptide hydrogel for localized chemotherapy prevents breast cancer recurrence. ACS Appl. Mater. Interfaces.

[143] Yiannopoulou K.G., Papageorgiou S.G. (2013). Current and future treatments for Alzheimer’s disease. Ther. Adv. Neurol. Disord..

[144] O’brien R.J., Wong P.C. (2011). Amyloid precursor protein processing and Alzheimer’s disease. Annu. Rev. Neurosci..

[145] Al Harthi S., Alavi S.E., Radwan M.A., El Khatib M.M., AlSarra I.A. (2019). Nasal delivery of donepezil HCl-loaded hydrogels for the treatment of Alzheimer’s disease. Sci. Rep..

[146] Chen W., Li R., Zhu S., Ma J., Pang L., Ma B., Du L., Jin Y. (2020). Nasal timosaponin BII dually sensitive in situ hydrogels for the prevention of Alzheimer’s disease induced by lipopolysaccharides. Int. J. Pharm..

[147] Zafar A., Alruwaili N.K., Panda D.S., Imam S.S. (2021). Potential of natural bioactive compounds in management of diabetes: Review of preclinical and clinical evidence. Curr. Pharmacol. Rep..

[148] Zhuang Y., Yang X., Li Y., Chen Y., Peng X., Yu L., Ding J. (2019). Sustained release strategy designed for lixisenatide delivery to synchronously treat diabetes and associated complications. ACS Appl. Mater. Interfaces.

[149] Wen N., Lü S., Xu X., Ning P., Wang Z., Zhang Z., Gao C., Liu Y., Liu M. (2019). A polysaccharide-based micelle-hydrogel synergistic therapy system for diabetes and vascular diabetes complications treatment. Mater. Sci. Eng. C.

[150] Guarner J., Brandt M.E. (2011). Histopathologic diagnosis of fungal infections in the 21st century. Clin. Microbiol. Rev..

[151] Bongomin F., Gago S., Oladele R.O., Denning D.W. (2017). Global and multi-national prevalence of fungal diseases-estimate precision. J. Fungus..

[152] Dudhipala N., Youssef A.A.A., Banala N. (2020). Colloidal lipid nanodispersion enriched hydrogel of antifungal agent for management of fungal infections: Comparative in-vitro, ex-vivo and in-vivo evaluation for oral and topical application. Chem. Phys. Lipids.

[153] Kumar M., Shanthi N., Mahato A.K., Soni S., Rajnikanth P.S. (2019). Preparation of luliconazole nanocrystals loaded hydrogel for improvement of dissolution and antifungal activity. Heliyon.

[154] AbouSamra M.M., Basha M., Awad G.E.A., Mansy S.S. (2019). A promising nystatin nanocapsular hydrogel as an antifungal polymeric carrier for the treatment of topical candidiasis. J. Drug Del. Sci. Tech..

[155] Van Gerwen O.T., Muzny C.A. (2019). Recent advances in the epidemiology, diagnosis, and management of Trichomonas vaginalis infection. F1000Research.

[156] Zhang Y., Miyamoto Y., Ihara S., Yang J.Z., Zuill D.E., Angsantikul P., Zhang Q., Gao W., Zhang L., Eckmann L. (2019). Composite thermoresponsive hydrogel with auranofin-loaded nanoparticles for topical treatment of vaginal trichomonad infection. Adv. Ther..

[157] Osmari B.F., Giuliani L.M., Reolon J.B., Rigo G.V., Tasca T., Cruz L. (2020). Gellan gum-based hydrogel containing nanocapsules for vaginal indole-3-carbinol delivery in trichomoniasis treatment. Eur. J. Pharm. Sci..

[158] Zaman K. (2010). Tuberculosis: A global health problem. J. Health Popul. Nutr..

[159] Anjani Q.K., Permana A.D., Cárcamo-Martínez Á., Domínguez-Robles J., Tekko I.A., Larrañeta E., Vora L.K., Ramadon D., Donnelly R.F. (2021). Versatility of hydrogel-forming microneedles in in vitro transdermal delivery of tuberculosis drugs. Eur. J. Pharm. Biopharm..

[160] Vashist A., Vashist A., Gupta Y.K., Ahmad S. (2014). Recent advances in hydrogel based drug delivery systems for the human body. J. Mater. Chem. B.

[161] Gu Z., Yu J., Zhang Y., Gallippi C. (2021). Thrombin-Responsive Hydrogels and Devices for Auto-Anticoagulant Regulation. U.S. Patent.

[162] Yang A.J.M., Domszy R.C., Yang J.C.H., Lynthera Corp (2020). High-Precision Drug Delivery by dual-Domain Ocular Device. U.S. Patent.

[163] Cull K. (2020). Treating Conditions Caused by Abnormal Growth of Pathogens in Body Cavities. U.S. Patent.

[164] Gutierrez C. (2020). Programmable Therapeutic Agent Delivery from Eye Mounted Device. U.S. Patent.

[165] Gutierrez C. (2020). Eye Mounted Device for Therapeutic Agent Release. U.S. Patent.

[166] Niven N. (2020). Medication Dispensing System. U.S. Patent.

[167] Herrero C., Ayoob A., Hanes J., Peris H. (2020). Apoptosis Inhibitor Formulations for Prevention of Hearing Loss. U.S. Patent.

[168] Naheed S. (2020). Medication. U.S. Patent.

[169] Ostrow Gregory I., Widder Kenneth J., Baker David S., Takruri H. (2020). Ophthalmic Composition. U.S. Patent.

[170] Florek C., Armbruster David A., Kerr Sean H., Jain S., Julien J., Bikram-Liles M. (2020). Biocompatible Organogel Matrices for Intraoperative Preparation of a Drug Delivery Depot. U.S. Patent.

[171] Yoon J., Kim K. (2012). Detecting signals of new technological opportunities using semantic patent analysis and outlier detection. Scientometrics.

[172] Trappey A.J., Trappey C.V., Wu C.Y., Lin C.W. (2012). A patent quality analysis for innovative technology and product development. Adv. Eng. Inform..

[173] Yoon J., Kim K. (2011). Identifying rapidly evolving technological trends for R&D planning using SAO-based semantic patent networks. Scientometrics.

[174] Chouhan D., Dey N., Bhardwaj N., Mandal B.B. (2019). Emerging and innovative approaches for wound healing and skin regeneration: Current status and advances. Biomaterials.

[175] Pourmoussa A., Gardner D.J., Johnson M.B., Wong A.K. (2016). An update and review of cell-based wound dressings and their integration into clinical practice. Ann. Transl. Med..

[176] New Hydroxyethyl Cellulose Hydrogel for the Treatment of the Pain of Knee Arthrosis. https://clinicaltrials.gov/ct2/results?cond=NCT04061733&term=&cntry=&state=&city=&dist=1.

[177] Ruptured Aneurysms Treated with Hydrogel Coils. https://clinicaltrials.gov/ct2/show/NCT03252314?cond=NCT03252314&draw=2&rank=1.

[178] Effect of the Negative Pressure Therapy Dressing Compared with Hydrogel Dressing. https://clinicaltrials.gov/ct2/show/NCT04265612?cond=NCT04265612&draw=2&rank=1.

[179] Comparative Clinical Performance of 59% Hioxifilcon a Contact Lenses vs. Marketed Hydrogel Contact Lens. https://clinicaltrials.gov/ct2/show/NCT04671108?cond=NCT04671108&draw=2&rank=1.

[180] Wound Treatment with 3% Sodium Pentaborate Pentahydrate. https://clinicaltrials.gov/ct2/show/NCT02241811?cond=NCT02241811&draw=2&rank=1.

[181] MucoLox Formulation to Mitigate Mucositis Symptoms in Head/Neck Cancer. https://clinicaltrials.gov/ct2/show/NCT03461354?cond=NCT03461354&draw=2&rank=1.

[182] Instylla HES Hypervascular Tumor Pivotal Study. https://clinicaltrials.gov/ct2/show/NCT04523350?cond=NCT04523350&draw=2&rank=1.

[183] Treatment of Knee Osteoarthritis with PAAG-OA. https://clinicaltrials.gov/ct2/show/NCT04045431?cond=NCT04045431&draw=2&rank=1.

[184] Clinical Performance of the Oxyaqua Daily Disposable Silicone Hydrogel Soft Contact Lens. https://clinicaltrials.gov/ct2/show/NCT03139201?cond=NCT03139201&draw=2&rank=1.

[185] Prostate-Rectal Separation with PEG Hydrogel and Its Effect on Decreasing Rectal Dose. https://clinicaltrials.gov/ct2/show/NCT02212548?cond=NCT02212548&draw=2&rank=1.

[186] Hydrogel Endovascular Aneurysm Treatment Trial. https://clinicaltrials.gov/ct2/show/NCT01407952?cond=NCT01407952&draw=2&rank=1.

[187] Clinical Performance of a Daily Disposable Toric Silicone Hydrogel Contact Lens. https://clinicaltrials.gov/ct2/show/NCT04464044?cond=NCT04464044&draw=2&rank=1.

[188] Intra-Articular PVA Hydrogel in Knee Osteoarthritis. https://clinicaltrials.gov/ct2/show/NCT04693104?cond=NCT04693104&draw=2&rank=1.

[189] TracelT Hydrogel in Localizing Bladder Tumors in Patients Undergoing Radiation Therapy for Bladder. https://clinicaltrials.gov/ct2/show/NCT03125226?cond=NCT03125226&draw=2&rank=1.

[190] Fitting Evaluation of Hydrogel and Silicone Hydrogel Sphere Design Contact Lenses. https://clinicaltrials.gov/ct2/show/NCT03098745?cond=NCT03098745&draw=2&rank=1.

[191] Performance of Toric Hydrogel Lenses Following a Refit with Toric Silicone Hydrogel Lenses for 1 Month. https://clinicaltrials.gov/ct2/show/NCT03835221?cond=NCT03835221&draw=2&rank=1.

[192] Hydrogel Coil Versus Bare Platinum Coil in Recanalization Imaging Data Study. https://clinicaltrials.gov/ct2/show/NCT01516658?cond=NCT01516658&draw=2&rank=1.

[193] Correa S., Grosskopf A.K., Lopez Hernandez H., Chan D., Yu A.C., Stapleton L.M., Appel E.A. (2020). Translational applications of hydrogels. Chem. Rev..

[194] Hunziker E., Spector M., Libera J., Gertzman A., Woo S.L.-Y., Ratcliffe A., Lysaght M., Coury A., Kaplan D., Vunjak-Novakovic G. (2006). Translation from research to applications. Tissue Eng..

[195] Yu A.C., Chen H., Chan D., Agmon G., Stapleton L.M., Sevit A.M., Tibbitt M.W., Acosta J.D., Zhang T., Franzia P.W. (2016). Scalable manufacturing of biomimetic moldable hydrogels for industrial applications. Proc. Natl. Acad. Sci. USA.

[196] Guidolin K., Zheng G. (2019). Nanomedicines lost in translation. ACS Nano.

[197] Savina I.N., Ingavle G.C., Cundy A.B., Mikhalovsky S.V. (2016). A simple method for the production of large volume 3D microporous hydrogels for advanced biotechnological, medical and environmental applications. Sci. Rep..

[198] Galante R., Pinto T.J., Colaço R., Serro A.P. (2018). Sterilization of hydrogels for biomedical applications: A review. J. Biomed. Mater. Res. Part B.

[199] Tomatsu I., Peng K., Kros A. (2011). Photoresponsive hydrogels for biomedical applications. Adv. Drug Del. Rev..

[200] Caló E., Khutoryanskiy V.V. (2015). Biomedical applications of hydrogels: A review of patents and commercial products. Eur. Polym. J..

[201] Silva D., de Sousa H.C., Gil M.H., Santos L.F., Oom M.S., Alvarez-Lorenzo C., Saramago B., Serro A.P. (2021). Moxifloxacin-imprinted silicone-based hydrogels as contact lens materials for extended drug release. Eur. J. Pharm. Sci..

[202] Shi X., Cantu-Crouch D., Sharma V., Pruitt J., Yao G., Fukazawa K., Wu J.Y., Ishihara K. (2021). Surface characterization of a silicone hydrogel contact lens having bioinspired 2-methacryloyloxyethyl phosphorylcholine polymer layer in hydrated state. Colloids Surf. B.

[203] Silva D., de Sousa H.C., Gil M.H., Santos L.F., Amaral R.A., Saraiva J.A., Salema-Oom M., Alvarez-Lorenzo C., Serro A.P., Saramago B. (2021). Imprinted hydrogels with LbL coating for dual drug release from soft contact lenses materials. Mater. Sci. Eng. C.

[204] Guo S., DiPietro L.A. (2010). Factors affecting wound healing. J. Dent. Res..

[205] Dabiri G., Damstetter E., Phillips T. (2016). Choosing a wound dressing based on common wound characteristics. Adv. Wound Caref..

[206] Stashak T.S., Farstvedt E. (2008). Topical wound treatments. Equine Wound Manag..

[207] Zhang F., Han X., Guo C., Yang H., Wang J., Wu X. (2021). Fibrous aramid hydrogel supported antibacterial agents for accelerating bacterial-infected wound healing. Mater. Sci. Eng. C.

[208] Ling Z., Chen Z., Deng J., Wang Y., Yuan B., Yang X., Lin H., Cao J., Zhu X., Zhang X. (2021). A novel self-healing polydopamine-functionalized chitosan-arginine hydrogel with enhanced angiogenic and antibacterial activities for accelerating skin wound healing. Chem. Eng. J..

[209] Cheng W., Wang M., Chen M., Niu W., Li Y., Wang Y., Luo M., Xie C., Leng T., Lei B. (2021). Injectable antibacterial anti-inflammatory molecular hybrid hydrogel dressing for rapid MDRB-infected wound repair and therapy. Chem. Eng. J..

[210] Yan L., Han K., Pang B., Jin H., Zhao X., Xu X., Jiang C., Cui N., Lu T., Shi J. (2021). Surfactin-reinforced gelatin methacrylate hydrogel accelerates diabetic wound healing by regulating the macrophage polarization and promoting angiogenesis. Chem. Eng. J..

[211] Shanmugapriya K., Kim H., Kang H.W. (2021). EGFR-conjugated hydrogel accelerates wound healing on ulcer-induced burn wounds by targeting collagen and inflammatory cells using photoimmunomodulatory inhibition. Mater. Sci. Eng. C.

[212] Xuan H., Wu S., Fei S., Li B., Yang Y., Yuan H. (2021). Injectable nanofiber-polysaccharide self-healing hydrogels for wound healing. Mater. Sci. Eng. C.

[213] Wang X., Wang Z., Fang S., Hou Y., Du X., Xie Y., Xue Q., Zhou X., Yuan X. (2020). Injectable Ag nanoclusters-based hydrogel for wound healing via eliminating bacterial infection and promoting tissue regeneration. Chem. Eng. J..

[214] Drury J.L., Mooney D.J. (2003). Hydrogels for tissue engineering: Scaffold design variables and applications. Biomaterials.

[215] Chapekar M.S. (2000). Tissue engineering: Challenges and opportunities. J. Biomed. Mater. Res..

[216] Ziadlou R., Rotman S., Teuschl A., Salzer E., Barbero A., Martin I., Alini M., Eglin D., Grad S. (2021). Optimization of hyaluronic acid-tyramine/silk-fibroin composite hydrogels for cartilage tissue engineering and delivery of anti-inflammatory and anabolic drugs. Mater. Sci. Eng. C.

[217] Karimi-Soflou R., Nejati S., Karkhaneh A. (2021). Electroactive and antioxidant injectable in-situ forming hydrogels with tunable properties by polyethylenimine and polyaniline for nerve tissue engineering. Colloids Surf. B.

[218] Ding H., Li B., Liu Z., Liu G., Pu S., Feng Y., Jia D., Zhou Y. (2021). Nonswelling injectable chitosan hydrogel via UV crosslinking induced hydrophobic effect for minimally invasive tissue engineering. Carbohydr. Polym..

[219] Maharjan B., Park J., Kaliannagounder V.K., Awasthi G.P., Joshi M.K., Park C.H., Kim C.S. (2021). Regenerated cellulose nanofiber reinforced chitosan hydrogel scaffolds for bone tissue engineering. Carbohydr. Polym..

[220] Wang L., Lu R., Hou J., Nan X., Xia Y., Guo Y., Meng K., Xu C., Wang X., Zhao B. (2020). Application of injectable silk fibroin/graphene oxide hydrogel combined with bone marrow mesenchymal stem cells in bone tissue engineering. Colloids Surf. A Physicochem. Eng. Asp..

[221] Serafin A., Murphy C., Rubio M.C., Collins M.N. (2021). Printable alginate/gelatin hydrogel reinforced with carbon nanofibers as electrically conductive scaffolds for tissue engineering. Mater. Sci. Eng. C.

[222] Yang Y., Ritchie A.C., Everitt N.M. (2021). Recombinant human collagen/chitosan-based soft hydrogels as biomaterials for soft tissue engineering. Mater. Sci. Eng. C.

[223] Wang P., Zhang W., Yang R., Liu S., Ren Y., Liu X., Tan X., Chi B. (2021). Biomimetic poly (γ-glutamic acid) hydrogels based on iron (III) ligand coordination for cartilage tissue engineering. Int. J. Biol. Macromol..

[224] Liu X., Liu S., Yang R., Wang P., Zhang W., Tan X., Ren Y., Chi B. (2021). Gradient chondroitin sulfate/poly (γ-glutamic acid) hydrogels inducing differentiation of stem cells for cartilage tissue engineering. Carbohydr. Polym..

